# Chemical characterization and speciation of the soluble fraction of Arctic PM_10_

**DOI:** 10.1007/s00216-024-05131-0

**Published:** 2024-01-16

**Authors:** Matteo Marafante, Stefano Bertinetti, Luca Carena, Debora Fabbri, Mery Malandrino, Davide Vione, Silvia Berto

**Affiliations:** Department of Chemistry, University of Turin, Via Pietro Giuria, 7, 10125 Turin, Italy

**Keywords:** Polar aerosol, Water-soluble fraction, Oxalate speciation, Iron speciation, Chemometric data treatment

## Abstract

The chemical composition of the soluble fraction of atmospheric particulate matter (PM) and how these components can combine with each other to form different species affect the chemistry of the aqueous phase dispersed in the atmosphere: raindrops, clouds, fog, and ice particles. The study was focused on the analysis of the soluble fraction of Arctic PM_10_ samples collected at Ny-Ålesund (Svalbard Islands, Norwegian Arctic) during the year 2012. The concentration values of Na^+^, K^+^, NH_4_^+^, Ca^2+^, Mg^2+^, Mn^2+^, Cu^2+^, Zn^2+^, Fe^3+^, Al^3+^, Cl^−^, NO_2_^−^, NO_3_^−^, SO_4_^2−^, PO_4_^3−^, formate, acetate, malonate, and oxalate in the water-soluble fraction of PM_10_ were determined by atomic spectroscopy and ion chromatography. Speciation models were applied to define the major species that would occur in aqueous solution as a function of pH (2–10). The model highlights that (i) the main cations such as Na^+^, K^+^, Mg^2+^, and Ca^2+^ occur in the form of aquoions in the whole investigated pH range; (ii) Cu^2+^, Zn^2+^, and, in particular, Fe^3+^ and Al^3+^ are mostly present in their hydrolytic forms; and (iii) Al^3+^, Fe^3+^, and Cu^2+^ form solid hydrolytic species that precipitate at pH values slightly higher than neutrality. These latter metals show interesting interactions with oxalate and sulfate ions, too. The speciation models were also calculated considering the seasonal variability of the concentration of the components and at higher concentration levels than those found in water PM extracts, to better simulate concentrations actually found in the atmospheric aqueous phase. The results highlight the role of oxalate as the main organic ligand in solution.

**Graphical Abstract:**

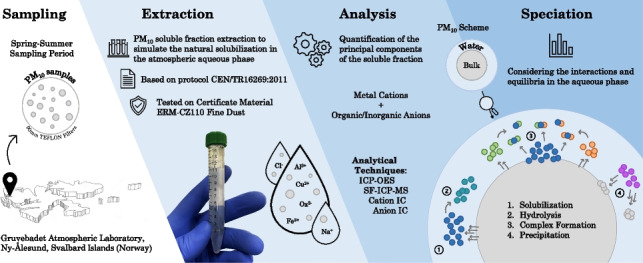

**Supplementary Information:**

The online version contains supplementary material available at 10.1007/s00216-024-05131-0.

## Introduction

Among the many constituents of the atmosphere, particulate matter (PM) is attracting the attention of the scientific community due to the various roles it plays in the air. PM impacts the surface energy budget [1, 2] and interacts with the coexisting gaseous and liquid phases. PM is present ubiquitously on Earth, from the big cities where it is principally produced by anthropic activity to the desert due to dust resuspension in air, passing through the inaccessible tropical forests or over the oceans [3]. From the isotopic study of Pb trapped in Greenland ice cores, it was reported the transport of PM from southern Europe already in antiquity [4]. Thanks to its geographic position, surrounded by many continents, the Arctic receives a great amount of PM from lower latitudes due to transport by the air masses: mineral dust, anthropogenic related substances, black carbon, and biogenic aerosol. It is suggested that mineral dust plays a major role in the faster temperature increase in the Arctic regions compared to lower latitudes, a phenomenon known as Arctic Amplification [2, 5]. On the other hand, mineral dust is often recognized as one of the main sources of some essential elements for remote ecosystems [6, 7]. For instance, the input of atmospheric Fe is very important for supporting the biological activity in the Arctic Ocean, which promotes CO_2_ sequestration from the atmosphere to the ocean waters [8]. To better understand the biogeochemical cycles of the elements, it is important to evaluate their distribution between solid phase and solution and to figure out which are the main species that can be formed. For instance, some metals such as Fe, Cu, and Mn can take part in redox reactions/photoreactions that are influenced by dissolved species; furthermore, the interaction with organic ligands can promote the solubilization of metals as in the case of oxalate with Fe [9–11]. Therefore, evaluating the speciation of the PM soluble fraction represents an interesting approach to understand the fate of the dissolved components and to hypothesize which species are effectively formed in solution. Finally, the study of the chemical processes occurring in the atmospheric liquid phase (clouds, raindrops, and liquid aerosol) presents interesting features: (i) homogenous chemical reactions in the liquid phase are usually faster than those in the gas phase, and some reactions can only take place in the liquid phase (e.g., reactions that involve ionic species); (ii) photochemical processes in solution are usually more efficient than those in air, and (iii) interactions between solid and liquid phases affect both heterogeneous reactions and the reactivity of the atmospheric liquid phase [12, 13].

This work reports on the concentrations of the main cationic — Na^+^, K^+^, NH_4_^+^, Ca^2+^, Mg^2+^, Mn^2+^, Cu^2+^, Zn^2+^, Fe^3+^, Al^3+^ — and anionic — Cl^−^, NO_2_^−^, NO_3_^−^, SO_4_^2−^, PO_4_^3−^, formate (hereafter, For), acetate (Ac), malonate (Mal), oxalate (Ox) — components in the water-soluble fraction of the Arctic PM_10_ collected in 2012 at the Svalbard Islands (Ny-Ålesund), describing them by principal component analysis (PCA). These data, together with the formation constants of the main species that can be derived by combination of the components, are then used to define the species that would actually occur in solution as a function of pH. As a first approximation, the formation constants used here were those reported at 298.15 K, because many formation equilibria have not yet been studied at lower temperature. The low concentrations of the ions detected in solution make the estimated ionic strength very low, allowing for the use of the thermodynamic formation constants in calculations (*T* = 298.15 K, *I* = 0 mol L^−1^).

The main goal of this work is to develop a method of investigation that provides new tools for the study of the interaction between the aerosol and the environment, with implications that go much beyond the studied 2012 PM_10_ samples. In fact, with the speciation models, it is possible to investigate the nature and the chemical behavior of the PM aqueous phase. This approach to the study of the composition of PM can provide information that ion analysis cannot give alone, including the hydrolytic behavior of a metal in solution or the formation of species with different features compared to the original components.

## Materials and methods

### Chemicals

Nitric acid (analytical grade, 65%; Merck KGaA, Darmstadt, Germany) was purified at sub-boiling (s.b.) grade by a PTFE distillation system (DST-1000 Acid Purification System, Savillex, Eden Prairie, MN, US). Water used for standards and sample preparation was of ultrapure grade (type 1) with 18.2 MΩ cm resistivity, produced by a Milli-Q system (Merk-Millipore, Darmstadt, Germany). Intermediate metal standard solutions were prepared from 1000 mg L^−1^ stock solutions (Sigma-Aldrich TraceCERT, Buchs, Switzerland). For the preparation of the cationic/anionic standards, the following chemicals at purity > 99% have been used: NH_4_Cl (Merck), NaAc (Carlo Erba, Cornaredo, Italy), NaFor (Merck), malonic acid (Aldrich), Na_2_Ox (Carlo Erba), NaNO_3_ (Sigma-Aldrich), Na_2_SO_4_ (Alfa Aesar, Kandel, Germany), Na_3_PO_4_·12H_2_O (Carlo Erba), and NaNO_2_ (Merck). For ion chromatography, KOH (Merck) and methanesulfonic acid (MSA, Sigma-Aldrich) were used for the preparation of the mobile phases. Fe(NO_3_)_3_·9H_2_O (Carlo Erba, for analysis grade) and K_2_Ox·H_2_O (Carlo Erba, ≥ 99.5%) were used for the voltammetric study of Fe^3+^-Ox system, using NaCl (Merck, for analysis grade) as ionic strength buffer and NaOH (Merck, ≥ 98%) to correct the pH of solutions.

### Sample collection and treatment

The PM_10_ samples investigated in this work have been collected on the roof of the atmospheric laboratory/observatory of Gruvebadet (GVB), about 800 m from the research facility of Ny-Ålesund (Svalbard Islands, 78°55′30″N, 11°55′40″E). Twenty-nine samples were collected during the boreal spring-summer (April–September) campaign of 2012. It was used a Tecora ECHO PM sampler, which employed 90-mm hydrophilic PTFE membrane filters (H100A090C) by Advantec MFS (Dublin, CA, USA), and was operated at a constant flow rate of 200 L min^−1^. After sampling, the PTFE filters were placed in pre-cleaned polyethylene Petri dishes, sealed in polyethylene bags, and stored at 253 K until analysis. The GVB laboratory is placed in a clear zone of radius = 500 m, in which any motorized activities are forbidden. Moreover, to avoid contamination of the samples by anthropic emissions from the village and the harbor of Ny-Ålesund or by the terrain surrounding the facility, an electronic system stops the sampling device in the rare cases in which the wind comes from Ny-Ålesund, or the wind velocity is < 0.5 m s^−1^.

One-half of each sample was dedicated to the extraction of the water-soluble fraction of PM_10_. The portion of filter was cut into four parts with ceramic scissors to facilitate their introduction into a same 15 mL polypropylene centrifuge tube. The extraction of the soluble fraction was performed using 15 mL of ultrapure water. The tubes were placed in a sonic bath for 30 min, checking that the temperature of the bath did not exceed 300 K [14]. After that, the samples were left to rest for 24 h before filtration with cellulose acetate syringe filters, pore size 0.45 μm (Merck-Millipore). The filtered solution was split into two portions. The first one was dedicated to the analysis of the cationic components, and it was acidified at 0.1% with sub-boiling HNO_3_. The remaining part was used for the analysis of anionic components. The aliquots thus obtained were stored at 277 K until analysis. To avoid contamination, due to the low concentration of the components, anything that enters in contact with the samples (scissors, plastic tweezers, tubes) was pre-cleaned with sub-boiling HNO_3_ solutions (0.1%) and ultrapure water. All the operations of sample manipulation were performed in a clean environment under a class-100 laminar flow hood.

### Instrumental analysis

The quantification of Al, Fe, Cu, Mn, Zn, Na, K, Mg, and Ca was performed by either ICP-OES (inductively coupled plasma-optical emission spectrometer) or HR-ICP-MS (high resolution-inductively coupled plasma-mass spectrometer), according to the concentration levels. In particular, the used ICP-OES was an Agilent 5110 (Santa Clara, CA, USA) simultaneous double-view instrument, equipped with a OneNeb nebulizer, a cyclonic spray chamber, and a VistaChip II CCD detector. HR-ICP-MS was a Thermo Fisher Scientific (Waltham, MA, USA) Element 2 instrument, equipped with a conical nebulizer, a baffled cyclonic spray chamber, a magnetic and electric sector, and a SEM detector. Wavelength, mass resolution, and isotope selection were optimized for each element, to avoid or minimize spectral interferences and to maximize sensitivity (Table S1 in the Online Resource).

Ammonium was analyzed with a Dionex DX 500 Ion Chromatograph, equipped with a Rheodyne injector (20 µL sample loop), LC-30 chromatography oven (set at 303 K), GP 40 gradient pump, Dionex Ion Pac CG12A (4 × 50 mm) guard column, Ion Pac CS12A (4 × 250 mm) cation exchange column, Thermo-Scientific CERS-5000 4 mm conductivity suppression unit, and ED-40 conductometric detector. The eluent was a 5.0×10^−6^ mol L^−1^ methanesulphonic acid solution at a flow rate of 1.0 mL min^−1^. The same instrument was used for the determination of anions, equipped with a Dionex IonPac AG19-HC guard column (4 × 50 mm), a Dionex IonPac AS19-HC column (4 × 250 mm), and an ASRS 300 electrochemical suppression unit. The chromatographic runs were performed in gradient mode using KOH in the concentration range of 1.2×10^−2^ mol L^−1^– 4.5×10^−2^ mol L^−1^ with a flow rate of 0.8 mL min^−1^.

Procedure blanks were prepared using ultrapure water and they followed the same sample preparation steps used for the real samples. The values of the blank’s concentrations were subtracted to the sample concentrations to eliminate contributions of sample manipulation.

The voltammetric study of the Fe^3+^-Ox complex was carried out with a PalmSens4 portable potentiostat (Thasar Srl, Milan, Italy), controlled by the software PSTrace 5.8. The electrochemical cells were composed of a glassy carbon electrode (GCE) as a working electrode, a platinum wire (0.5 mm, surface area of about 0.7 cm^2^) as a counter electrode, and a RE-1B Ag/AgCl, 3 mol L^−1^ KCl as a reference electrode. The pH of solutions was measured by a Methrom 713 potentiometer equipped with a combined glass electrode (Ag/AgCl/3M KCl internal reference).

### Data processing

Principal component analysis (PCA) was performed to better visualize the information hidden in the multivariate dataset composed of 19 variables for each of the 29 samples. PCA allows for representing data from multivariate space with a lower number of new variables, called principal components (PC), which are formed by linear combination of the original variables. The principal components are sorted according to the fraction of explained variance of the data. Therefore, the first components bring most of the useful information, excluding noise and spurious data. By means of PCA, it is possible to study relationships among samples and to highlight the variables that mostly affect the observed relationships [15].

To define the speciation models, alongside the concentrations of the ions in each sample, it is necessary to define the formation constants of the relevant species formed by interaction of the ions. Moreover, hydrolytic species of the cations and dissociation constants of the protogenic components were also considered in the model. The complete set of formation and solubility constants is given in Tables S2–S4. These constants were derived from the literature and, whenever possible, they referred to quite low ionic strength, because of the low concentration of the ionic components in the studied samples. If necessary, the extended Debye-Hückel equation (EDH, Eqs. 1–5; [16]) was applied to the literature values, to obtain the thermodynamic constants log*K* and log *β* at ionic strength *I*=0 mol L^−1^ and *T* = 298.15 K [17]. In fact, EDH can be used to define the value of a formation constant at a different value of *I* but at the same temperature, if the original value at a specific *I′* and *T* is known. Because of the scarce interaction between the investigated anions and the alkaline cations, whenever possible, the selected constants were those obtained with non-interacting cations. The thermodynamic constants thus defined were used to calculate the distribution diagram of the species by the open-source software PyES [18]. This software considers the variation of the ionic strength of the medium when varying the pH, due to the variation of the charged species in solution, and it corrects the formation constant by applying the EDH equation:

1 $$log{K}_{i}\left(I\right)=log{K}_{i}\left(I^{\prime}\right)-{z}^{*}A\left(\frac{\sqrt{I}}{1+B\sqrt{I}}-\frac{\sqrt{{I}^{\prime}}}{1+B\sqrt{{I}^{\prime}}}\right)+{C}_{i}\left(I-{I}^{\prime}\right)+{D}_{i}\left({I}^{\frac{3}{2}}-{I^{\prime}}^{\frac{3}{2}}\right)$$

2 $${C}_{i}={c}_{0}{{p}^{*}}_{i}+{c}_{1}{{z}^{*}}_{i}$$

3 $${D}_{i}={d}_{o}{{p}^{*}}_{i}+{d}_{1}{{z}^{*}}_{i}$$

4 $${{p}^{*}}_{i}=\sum {p}_{reactants}-\sum {p}_{products}$$

5 $${{z}^{*}}_{i}=\sum {z}_{reactants}^2-\sum {z}_{products}^2$$

where *p* and *z* are, respectively, the stoichiometric coefficients and the charges of the involved species. By considering a non-interacting medium in solution, with Na and K as components of the speciation model and not only as background ions, the values *A* = 0.5, *B* = 1.5, *c*_0_ = 0.1, *c*_1_ = 0.23, *d*_0_ = 0, and *d*_1_ = −0.1 can be used at *I* ≤ 1.0 mol L^−1^ and *T* ≤ 318.15 K [16, 18].

## Results and discussion

### Concentrations of the main components

**Fig 1:**
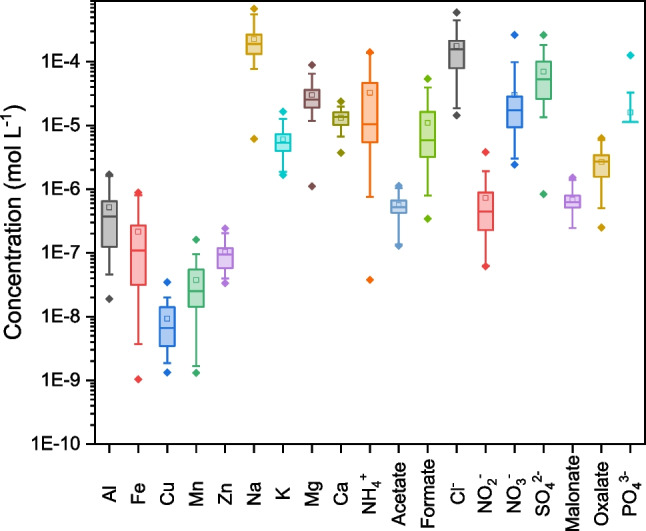
Box plot of components’ concentration, in the soluble fraction extracted from the Arctic PM_10_ samples. The squares indicate the mean values*,* and the whiskers are determined by the 5th and 95th percentiles of each distribution. Dots represent minimum and maximum values, respectively

The concentration values and the associated uncertainties of the main components of the soluble fraction of PM_10_ samples collected at Ny-Ålesund during the spring–summer campaign of 2012 are reported in Table S5 and S6 in the Online Resource, and are graphically shown in Fig. 1. The amount of the components is expressed as mol L^−1^ in the solution obtained by extraction of the soluble fraction. Considering that the volume of air collected (*V*_*i*_) varied among the samples, the concentrations have been corrected in order to be all referred to the average volume of sampled air ($\overline{V}$) using a multiplicative correction factor *f*_*i*_ (Eq. 6).

6 $${f}_{i}=\frac{\overline{V}}{{V }_{i}}$$

The determined components were as follows: Al, Fe, Cu, Mn, and Zn by ICP-MS; Na, K, Mg, and Ca by ICP-OES; NH_4_^+^, F^−^, Ac, For, Cl^−^, NO_2_^−^, NO_3_^−^, Br^−^, SO_4_^2−^, Mal, Ox, and PO_4_^3−^ by ion chromatography. Fluoride and bromide were under the limits of detection (LOD) of the technique for all the samples and were no longer taken into consideration. Differently, phosphate was considered for the definition of the speciation model because of its ability to act as ligand, even if it occurred at low concentration in many samples. In the case of concentration values below the LOD (Table S7), the missing data were replaced with values estimated by principal component analysis (PCA). When it was not possible to operate in this way (too few available data to perform PCA or negative value(s) coming from reconstruction processes), the missing data were substituted with the LOD. The LOD values for the different techniques were estimated by the calibration curves, as 3 times the residual standard *deviation s*_*x/y*_ divided by the slope of the regression curve [19]. Satisfying recoveries were obtained from the analysis of certified reference materials if available (Table S8), with error values better than ±10% excluding phosphate (−27%). For this reason, the concentration of phosphate must be considered as only indicative. In Table 1, a statistical summary of components’ concentration is reported. The concentrations obtained by the used extraction procedure are similar to those that can be recorded in precipitation collected in many sites around the Arctic (Table S9).

**Table 1:** Statistical summary of the components’ concentration: mean, standard deviation (s), relative standard deviation (RSD), median, inter-quartile range (IQR), median absolute deviation (MAD), and relative median absolute deviation (RMAD)

Component	Mean (mol L^−1^)	s (mol L^−1^)	RSD (%)	Median (mol L^−1^)	IQR (mol L^−1^)	MAD (mol L^−1^)	RMAD (%)
Al	5.2×10^−7^	5.2×10^−7^	100	3.7×10^−7^	5.2×10^−7^	2.7×10^−7^	74
Fe	2.2×10^−7^	2.6×10^−7^	122	1.1×10^−7^	2.4×10^−7^	8.9×10^−8^	81
Cu	9.3×10^−9^	7.6×10^−9^	82	6.7×10^−9^	1.1×10^−8^	3.8×10^−9^	56
Mn	3.7×10^−8^	3.6×10^−8^	97	2.5×10^−8^	4.1×10^−8^	1.8×10^−8^	72
Zn	1.0×10^−7^	5.5×10^−8^	54	9.4×10^−8^	6.1×10^−8^	3.6×10^−8^	39
Na	2.3×10^−4^	1.5×10^−4^	65	1.9×10^−4^	1.3×10^−4^	7.0×10^−5^	37
K	6.1×10^−6^	3.3×10^−6^	54	5.4×10^−6^	3.3×10^−6^	1.6×10^−6^	30
Mg	3.0×10^−5^	1.8×10^−5^	59	2.5×10^−5^	1.7×10^−5^	8.0×10^−6^	31
Ca	1.3×10^−5^	4.5×10^−6^	34	1.4×10^−5^	5.9×10^−6^	2.7×10^−6^	20
NH_4_^+^	3.3×10^−5^	4.1×10^−5^	125	1.0×10^−5^	4.1×10^−5^	8.9×10^−6^	85
Ac	5.6×10^−7^	2.4×10^−7^	42	5.2×10^−7^	2.3×10^−7^	1.2×10^−7^	23
For	1.1×10^−5^	1.2×10^−5^	109	5.9×10^−6^	1.3×10^−5^	3.8×10^−6^	65
Cl^−^	1.8×10^−4^	1.4×10^−4^	79	1.6×10^−4^	1.3×10^−4^	7.8×10^−5^	49
NO_2_^−^	7.3×10^−7^	7.9×10^−7^	109	4.5×10^−7^	6.6×10^−7^	3.8×10^−7^	85
NO_3_^−^	3.0×10^−5^	5.0×10^−5^	163	1.7×10^−5^	1.9×10^−5^	1.0×10^−5^	58
SO_4_^2−^	7.0×10^−5^	5.8×10^−5^	84	5.3×10^−5^	7.4×10^−5^	3.0×10^−5^	56
Mal	6.9×10^−7^	3.2×10^−7^	46	6.3×10^−7^	2.8×10^−7^	1.7×10^−7^	26
Ox	2.7×10^−6^	1.6×10^−6^	59	2.7×10^−6^	1.9×10^−6^	9.2×10^−7^	34
PO_4_^3−^	1.6×10^−5^	2.2×10^−5^	135	1.1×10^−5^	0	0	0

### Multivariate analysis

Multivariate techniques are key tools to handle information hidden in a complex dataset. Among these instruments, the PCA is one of the most used in source recognition based on chemical characterization of PM_10_ [20]. For this analysis, some variables (in particular, Ac and Mal) were not considered because almost half of their values were under the LOD. For the other variables, missing data were left undefined. Before statistical treatment, data were autoscaled to allow for comparison between variables with different magnitudes.

Table 2 reports the loading values for the first three PCs, which altogether describe almost 82% of the total variance of the whole dataset. The loadings are the weights of the variables in the linear combination that defines each PC: the higher the absolute value of the loading, more the PC is described by that variable. The most significant loading values for each PC are indicated in italic font. So, PC1 describes the amount of Al, Fe, Cu, Mn, and Zn, but also NH_4_^+^ and SO_4_^2-^, which can be connected to crustal sources or anthropogenic activity [21]. PC2 is linked with the chemical components of marine origin, that is, Na, K, Mg, and Cl^−^ [22]. Finally, PC3 describes the amount of For and NO_3_^−^. This information is supported by Pearson’s correlation coefficients reported in Table S10 of the Online Resource.

**Table 2:** Loading values for the first three principal components (PC1, PC2, and PC3) and the amount of the total variance of the data that is explained by each component

Chemical component	PC1 (37.5%)	PC2 (28.3%)	PC3 (12.5%)
Al	*0.369*	0.155	0.015
Fe	*0.361*	0.139	0.073
Cu	*0.308*	0.030	0.001
Mn	*0.342*	0.105	0.097
Zn	*0.305*	0.147	0.119
Na	0.077	*−0.451*	0.035
K	0.151	*−0.425*	0.008
Mg	0.095	*−0.454*	0.047
Ca	0.254	−0.281	0.119
NH_4_^+^	*0.359*	0.167	0.051
For	0.102	−0.007	*−0.634*
Cl^−^	−0.081	*−0.422*	0.104
NO_2_^−^	0.143	−0.171	−0.166
NO_3_^−^	0.015	−0.042	*−0.647*
SO_4_^2−^	*0.345*	−0.122	0.029
Ox	0.203	−0.043	−0.302

**Fig. 2:**
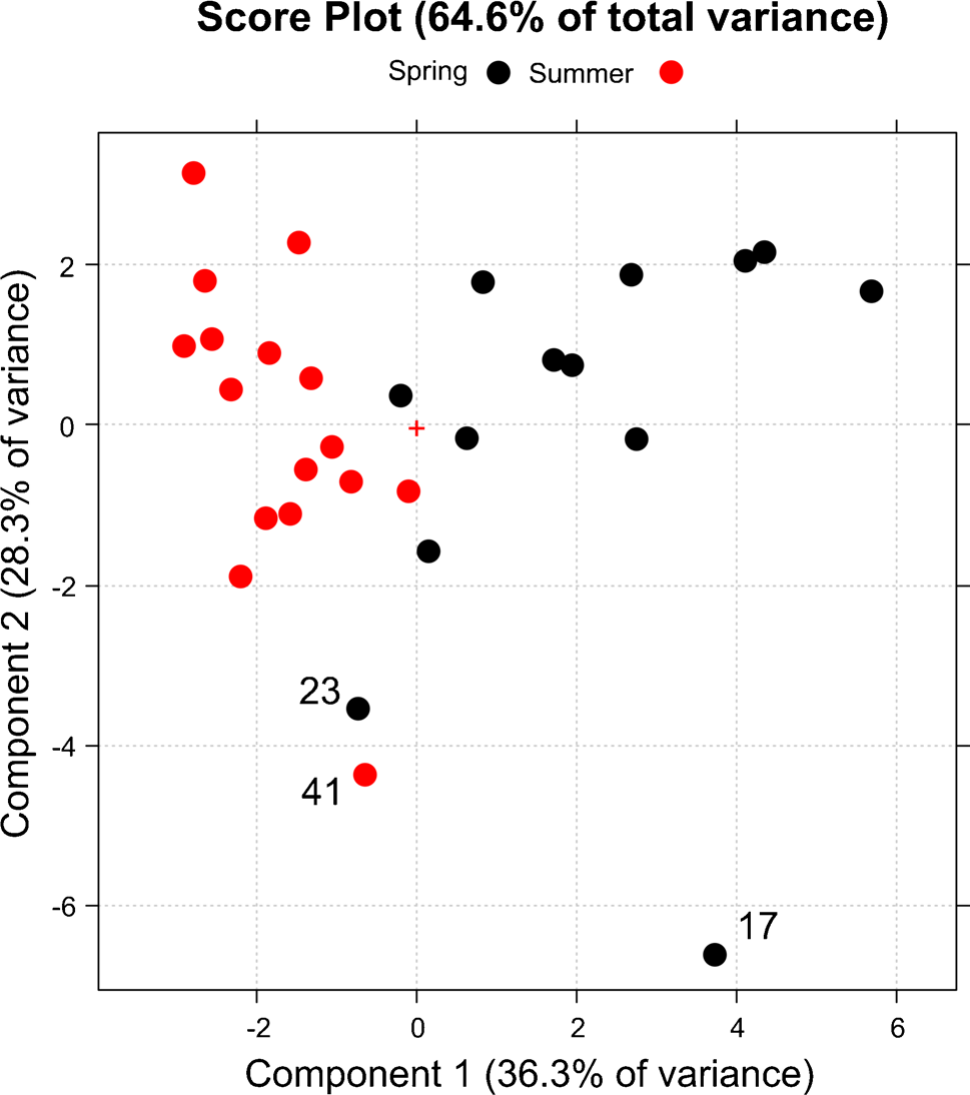
Score plot for PC1 and PC2. The PM_10_ samples are grouped into two classes, Spring (black points) and Summer (red points), according to the date in which they were collected

Figure 2 shows the score plot for the first two principal components. The samples are grouped into two classes, named “spring” and “summer” according to the date in which they were collected. The 21st of June is used as the watershed date between them. It emerges how PC1 can separate the two classes: the spring samples fall at positive values (their mean score value on PC1 is 2.11) and those of the summer class at negative values (mean scores on PC1 of −1.72). Moreover, the spring samples showed higher dispersion along the PC1 (standard deviation 1.97) than the summer samples (standard deviation 0.82), highlighting a higher variability in the amount of Al, Fe, Cu, Mn, Zn, NH_4_^+^, and SO_4_^2−^ in spring. The PC2 is highly affected by the chemical components related to the marine source, and the samples labeled with 17, 23, and 41 are those with higher amounts of these components. Moreover, Na, K, Mg, and Cl^−^ do not show clear differences between the two classes.

### Sea salt and non-sea salt contributions for Na, Ca, and sulfate

As previously reported in other studies [23, 24], the origin of calcium and sulfate in Arctic PM_10_ is often related to the synchronous activity between the marine source and, respectively, crustal and anthropogenic sources. This aspect is confirmed by looking at the loading score (Fig S1 in the Online Resource), in which the variables Ca and SO_4_^2−^ fall apart from the cluster of the marine chemical component.

Here, the partition between the sea salt and non-sea salt contributions for Na, Ca, and SO_4_^2−^ was calculated resolving the following system of linear equations [25]:

7 $$tot{\text{Na}}=ss{\text{Na}}+nss{\text{Na}}$$

8 $$tot{\text{Ca}}=ss{\text{Ca}}+nss{\text{Ca}}$$

9 $$tot{{\text{SO}}}_{4}^{2-}=ss{{\text{SO}}}_{4}^{2-}+nss{{\text{SO}}}_{4}^{2-}$$

10 $$ss{\text{Na}}=tot{\text{Na}}-0.980 nss{\text{Ca}}$$

11 $$nss{\text{Ca}}=tot{\text{Ca}}-0.022 ss{\text{Na}}$$

12 $$nss{{\text{SO}}}_{4}^{2-}=tot{{\text{SO}}}_{4}^{2-}-0.061 ss{\text{Na}}$$

where totX is the total concentration (mol L^−1^) of component X in the solution, ssX is the concentration of component X due to the sea-salt contribution, and nssX is the concentration due to the non-sea salt contribution. The coefficient 0.980 is the molar ratio between Na and Ca in the crust, 0.022 is the molar ratio of Ca and Na in seawater, and 0.061 is the molar ratio between SO_4_^2−^ and Na in seawater [26]. The results are reported in Fig S2. As expected, Na had mainly marine origin (mean ssNa = 93%) in both spring and summer samples, as previously observed by PCA. A significant non-sea salt contribution for Na (54%) was only observed in the sample collected between 24/06/2012 and 28/06/2012. In contrast, Ca showed a predominant non-sea salt contribution (mean nssCa = 65%) in all the samples. Finally, SO_4_^2−^ had significant seasonality with the sea-salt fraction increasing its contribution from spring to summer, in parallel with the decrease of total concentration. These trends can be ascribed to the anthropogenic emissions that are an important source of S to the atmosphere (especially as SO_2_), and that are more active during the cold months [14]. Moreover, the high stability of the Arctic troposphere during cold months promotes the accumulation and long-range transport of anthropic emission-related substances [27].

Sulfuric and nitric acids are the most important acidic species usually present in the atmosphere, respectively produced upon oxidation of SO_2_ and NO_*x*_. The good balance between the total equivalents of positive and negative charged species in the aqueous PM_10_ extracts (Fig S3) suggests that SO_4_^2−^ and nitrate are predominantly neutralized by ammonium ions, with NH_3_ being the main basic species in the particulate matter [28].

### Speciation of the main components

**Fig. 3:**
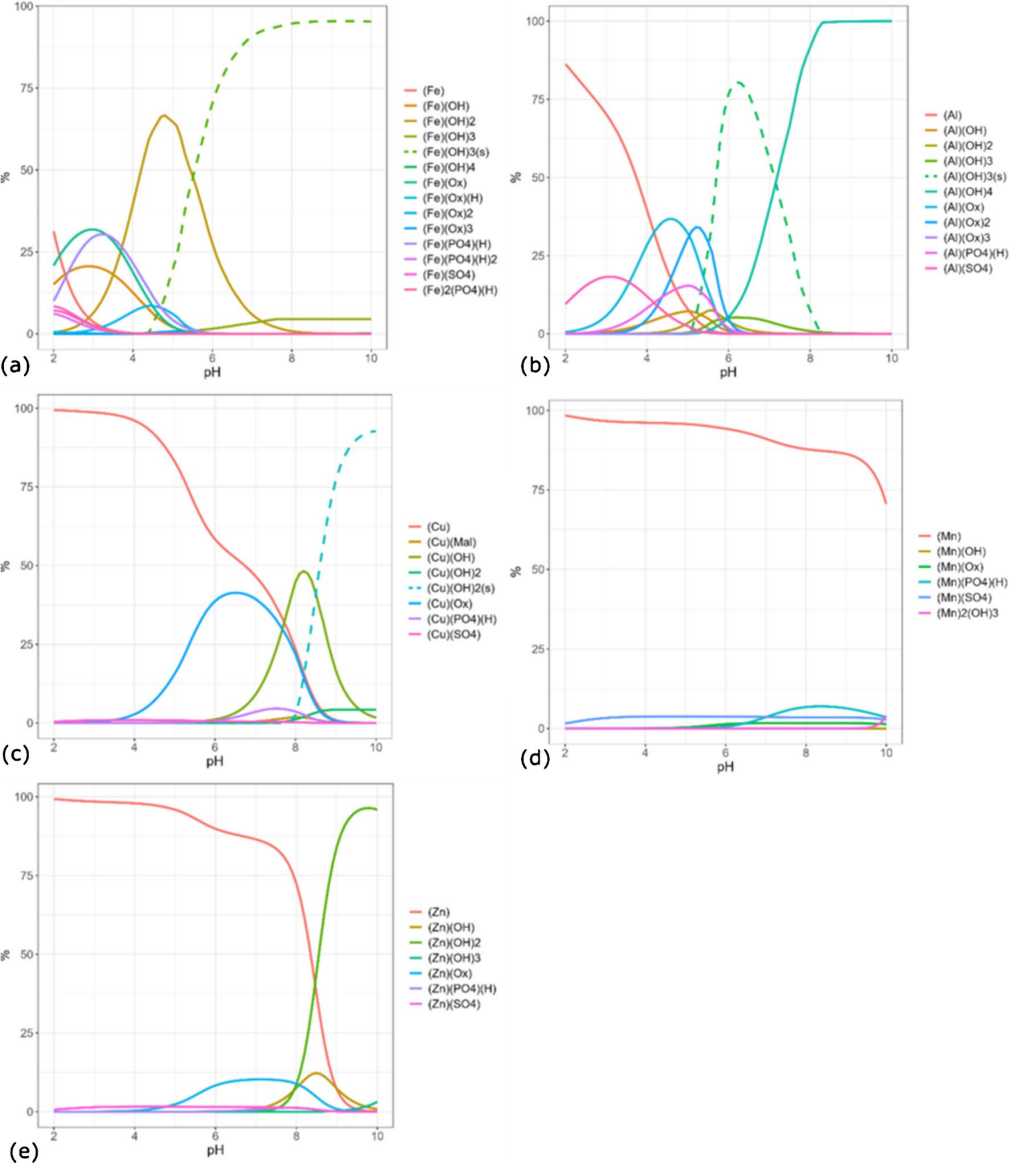
Mean species distribution diagrams of (**a**) Fe^3+^, (**b**) Al^3+^, (**c**) Cu^2+^, (**d**) Mn^2+^, and (**e**) Zn^2+^ as a function of pH, for the soluble fraction of 29 Arctic PM_10_ samples collected in 2012

Using the speciation model described above and the concentrations of the components in solution, the distribution diagram of the main species as a function of pH can be obtained (see Fig 3 and S4). The speciation model was applied considering the main components of the soluble fraction of the Arctic PM_10_. Therefore, even if the distribution diagrams were calculated between pH 2 and 10, the sub-range 4–6 is most realistic with respect to the pH values recorded in precipitations in the Arctic area (Table S9) [29–31]. To describe a representative system of the soluble fraction of Arctic PM_10_, the results here reported are referred to the average diagram distribution obtained by mediating the speciation models calculated individually for the 29 samples. The metals Mn, Cu, Zn, Fe, and Al are considered in their most common oxidation state for the calculation of speciation model: (II), (II), (II), (III), and (III), respectively. Alkaline and earth-alkaline metals are considered in their highest oxidation states.

The distribution diagrams in Fig S4 for Na^+^, K^+^, and Mg^2+^ showed that these metals occur predominantly as aquoions (also reported as *free metal*) at all investigated pH values, as already reported previously in the speciation of Antarctic atmospheric depositions [17]. Differently, Ca^2+^ starts being linked to HPO_4_^2−^ and PO_4_^3−^ at pH > 7, with [CaPO_4_]^−^ gaining sharp importance from pH > 8 (Fig S4 of Online Resource). Regarding the other metallic components (Fig 3), Fe^3+^ predominantly occurred as the hydrolytic form [Fe(OH)_2_]^+^ in the pH range 4–6, with a lower but significant amount of iron engaged to form species with oxalate ([Fe(Ox)]^+^ and [Fe(Ox)_2_]^−^) and phosphate ([Fe(HPO_4_)]^+^), especially at pH < 4. The precipitation of Fe(OH)_3 (s)_ starts above pH ≈ 4.5 (dashed curve in Fig. 3a). Similarly, Al^3+^ forms abundant species with oxalate, [Al(Ox)]^+^ and [Al(Ox)_2_]^−^, and phosphate ([Al(HPO_4_)]^+^). In this case, however, the abundance maxima of these species fall in the pH range 4–6. The amount of free Al^3+^ is negligible from around pH 6 where, instead, most of the aluminum precipitates as Al(OH)_3 (s)_. At higher pH values, the solid is no longer thermodynamically stable and [Al(OH)_4_]^−^ becomes the most abundant Al species in solution (Fig. 3b). Copper occurs as free metal in solution up to pH 4, above which the formation of the Cu(Ox) complex with oxalate significantly increases and reaches the maximum between pH 6 and 7. At higher pH values, Cu^2+^ occurs as hydrolytic form [CuOH]^+^ and solid Cu(OH)_2 (s)_ (Fig. 3c). The behavior of Mn^2+^ is quite different, because the free metal species still predominates at pH values up to 8 (Fig. 3d). Finally, also Zn^2+^ occurs as free metal at pH<8, with a scarce formation of a complex with oxalate (Zn(Ox), accounting for around 10% of total Zn) in the pH range 6–8. At higher pH values, the free species is completely converted into the hydrolytic forms [ZnOH]^+^ and Zn(OH)_2_ (Fig. 3e). Among the investigated ligands, oxalate turns out to be the most important due to its ability to form stable species with Fe^3+^, Al^3+^, Cu^2+^, and Zn^2+^ in the pH range 4–6. Sulfate is the second most important ligand, while the others do not show any remarkable effect. As already reported in the speciation study of Antarctic deposition [17], Fe^3+^ is present in solution as hydrolytic forms or linked with oxalate and phosphate at low pH, differently from the other metals for which the free forms (aquoions) are predominant.

The atmospheric composition over the Arctic region changes significantly over the year, due to strong variability in the environmental conditions (atmospheric stability, temperature, sunlight irradiation) among the seasons [32, 33]. As reported above, this variability affects the concentration of some components and, therefore, their speciation. In fact, the concentrations of the components and their relative differences determine which species will be formed in solution and their respective importance. Therefore, the difference in the mean species’ distribution diagram for the spring and summer samples was also studied. Significant differences were observed for Al^3+^, Cu^2+^, and Fe^3+^, for which the formation of species with sulfate and oxalate is higher in spring than in summer. In contrast, hydrolytic forms, free metals, and complexes with HPO_4_^2−^ are enhanced in the summer months. Differently, the precipitation of Al(OH)_3_, Cu(OH)_2_, and Fe(OH)_3_ is enhanced in spring (Fig S5) due to higher metal concentrations.

PM particles in the air play an important role as cloud condensation nuclei (CCN), promoting the condensation of water molecules on them, the subsequent dissolution of the soluble fraction, and the formation of many dissolved species. The results reported so far considered the use of 15 mL of water for the extraction of the soluble fraction of PM_10_. Considering that the average volume of sampled air is 1050 m^3^, and assuming that all the 15 mL water volume is in the air as aerosol droplets, an aerosol liquid water content (ALWC) of about 14.2 mg m^−3^ is obtained. This value is about 100–1000 times higher than found in subtropical and continental regions [34, 35]. Moreover, in polar regions, the low temperature significantly reduces the air humidity. By using the software ISORROPIA II [36], the ALWC can be simulated from the main ions detected in the aerosol (Na^+^, K^+^, Ca^2+^, Mg^2+^, NO_3_^−^, Cl^−^, SO_4_^2−^), the temperature (*T*), and the relative humidity (HR). Using the median values of ions concentrations in the samples, the median *T* (268.75 K), and the relative HR (67.3%) at Ny-Ålesund during the sampling campaign of 2012 [37], an ALWC value of 0.26 μg m^−3^ was obtained. Therefore, in the obtained water extracts, the concentrations of the components in the aerosol liquid phase were severely underestimated. In the same way, also the aerosol water layer can reach pH < 4. New speciation models, with the components’ concentrations increased by a factor of 10,000, have thus been calculated, exploiting the ability of PyES to recalculate the input thermodynamic constants at the new ionic strength conditions using the EDH equation. This extrapolation is affected by high uncertainty, because the EDH equation was tested to give good approximation of the stability constants for I ≤ 1.0 mol L^−1^, whereas the median ionic strength of our solutions would be 1.9 mol L^−1^. Therefore, the simulation provides only an approximate description of the chemical system. In these extremely concentrated solutions, the speciation models for Na^+^, K^+^, and Mg^2+^ show an increment of the interaction with chloride and sulfate, which strongly reduces the amount of free metals. Moreover, more than 95% of Ca^2+^ occurs as solid CaSO_4_ over the entire pH range. About the other metals, especially in the cases of Al^3+^, Cu^2+^, and Fe^3+^, one observes an increase in the interaction with oxalate. Finally, the predominance of Fe-oxalate species, [Fe(Ox)_2_]^−^ and [Fe(Ox)_3_]^3−^, is prominent up to pH 5, above which the precipitation of Fe(OH)_3_ sharply increases (Fig S6). Therefore, it seems that Fe-oxalate complexes replace in importance the hydrolytic forms at pH 4–5 in concentrated solutions.

Due to the important role of Ox that emerged from chemical modeling, a preliminary voltammetric study was performed with a simplified system to experimentally confirm the interaction between Fe^3+^ and Ox predicted by the speciation models. Cyclic voltammetry experiments (CV) were registered in solutions containing Fe^3+^ and Ox at high concentrations (in the range of 0.002 mol L^−1^–0.02 mol L^−1^) in NaCl 1.0 mol L^−1^, and at different pH values (Fig S7). The composition was chosen on the basis of the concentration ratios experimentally detected in real samples, and new speciation models were built up with the same concentrations used in the voltammetric studies (Fig S8). The voltammetric results can be explained by the information that emerged from the speciation models. Thus, the shift in the oxidation potential when varying the pH (Fig S7b) can be justified considering the change of the main Fe^3+^-Ox species (Fig S8d) moving from pH 1 (where [Fe(Ox)_2_]^−^ predominates) to pH 4 (where [Fe(Ox)_3_]^3−^ predominates). Moreover, the rise in the current observed when increasing the amount of Ox (Fig S7c) can be justified by the capability of the ligand to avoid the precipitation of Fe^3+^, by forming the dissolved species [Fe(Ox)_3_]^3−^ as predicted by the speciation models shown in Fig S8.

## Conclusions

In this work, a first approximation of the speciation of the main metals occurring in the soluble fraction of Arctic PM_10_ has been proposed. The concentration of the investigated metals reflects their origin and, understandably given the sampling location, metals associated with the marine source (Na, K, Mg, and Ca) reached higher concentrations. The other components, associated with crustal and anthropogenic sources, often had lower concentrations but showed seasonal variability. All these aspects impact the speciation of dissolved metals. The speciation models suggest an important role of oxalate as ligand for stabilizing Al^3+^, Fe^3+^, and Cu^2+^ in solution. The higher metals’ concentration in the spring samples promotes the formation of species with sulfate and oxalate and the precipitation of hydrolytic species, while soluble hydrolytic species are enhanced in summer. Because of the low humidity in the cold air masses of the Arctic, the amount of water available for the solubilization of PM_10_ ions is quite lower than that used for the extraction procedure. As a consequence, when PM acts as CCN, it would be covered by a very concentrated water layer. By increasing the components’ concentration by a factor of 10,000, the role of oxalate as main ligand agent was further highlighted. Preliminary experimental studies conducted on concentrated solutions of a simplified Fe^3+^-Ox system have confirmed the predictions of the speciation models.

As reported previously, these must be considered as preliminary results that are useful to define the main species that could be formed in solution. In fact, the thermodynamic constants used here for the speciation models are referred to 298.15 K, a temperature that is far away from those found at the Svalbard. To improve the modeling capacity, future studies will thus be necessary to estimate the formation constants of these species, together with the related protonation constants of the ligands, at lower temperatures and different ionic strengths.

## Supplementary Information

Below is the link to the electronic supplementary material.

Supplementary file1 (DOCX 5783 KB)
