# Supplementary material for: Chemical characterization and speciation of the soluble fraction of Arctic PM10
Source: Anal Bioanal Chem. 2024 Jan 16;416(6):1389–98. doi: 10.1007/s00216-024-05131-0 (PMC11461546; doi:10.1007/s00216-024-05131-0)
Supplement: Supplementary file 1 — Supplementary file1 (DOCX 5783 KB) [file 216_2024_5131_MOESM1_ESM.docx]

Supplementary Material

Analytical and Bioanalytical Chemistry

Chemical characterization and speciation of the soluble fraction of Arctic PM_10_

# Matteo Marafante^1^, Stefano Bertinetti^1*^, Luca Carena^1^, Debora Fabbri^1*^, Mery Malandrino^1^, Davide Vione^1^, Silvia Berto^1^

^1^ Department of Chemistry, University of Turin, Via Pietro Giuria, 7 – 10125 Torino (Italy)

* stefano.bertinetti@unito.it, debora.fabbri@unito.it

Table S1. Elements analysed in this work, the instrumentation used, and the instrumental conditions adopted.

| **Element** | **Instrument** | **Wavelength (nm) /**  **Isotope (a.m.u.) – Resolution** |
| --- | --- | --- |
| Na | ICP-OES ^a^ | 589.592 |
| K | ICP-OES | 766.491 |
| Mg | ICP-OES | 279.553 |
| Ca | ICP-OES | 396.847 |
| Fe | HR-ICP-MS ^b^ | 56 – MR ^c^ |
| Cu | HR-ICP-MS | 63, 65 – MR |
| Mn | HR-ICP-MS | 55 - MR |
| Zn | HR-ICP-MS | 64, 66, 68 - LR ^d^ |
| Al | HR-ICP-MS | 27 - LR |

^a^ ICP-OES: Inductively Coupled Plasma-Optical Emission Spectrometer; ^b^ HR-ICP-MS: High Resolution-Inductively Coupled Plasma-Mass Spectrometer ^c^ MR: medium resolution; ^d^ LR: low resolution

Table S2. Stepwise (logK) and overall (logβ) protonation constant of the protogenic species.

| **Protonation constants** | | | | | |
| --- | --- | --- | --- | --- | --- |
| **Species** | **log*K ^a^*** | **log*β ^b^*** | **Ref.** | **log*K*** | **log*β*** |
|  | **Original values** | |  | **Values estimated by the application of EDH equation** [1] | |
|  | *I = 0 mol L^-1^, T = 298.15 K* | |  | *I = 0 mol L^-1^, T = 298.15 K* | |
| HSO_4_^-^ | 1.987 | 1.987 | [2] | 1.987 | 1.987 |
|  | *I* = 0 mol L^-1^, *T = 298.15 K* | |  | *I = 0 mol L^-1^, T = 298.15 K* | |
| HPO_4_^2-^ | 12.35 | 12.35 | [3] | 12.35 | 12.35 |
| H_2_PO_4_^-^ | 7.20 | 19.55 |  | 7.20 | 19.55 |
| H_3_PO_4_ | 2.15 | 21.70 |  | 2.15 | 21.70 |
|  | *I = 0 mol L^-1^, NEt_4_I^c^, T = 298.15 K* | |  | *I = 0 mol L^-1^, T = 298.15 K* | |
| HAc ^d^ | 4.74 | 4.74 | [4] | 4.74 | 4.74 |
|  | *I = 0.16 mol, NEt_4_I, L^-1^, T = 298.15 K* | |  | *I = 0 mol L^-1^, T = 298.15 K* | |
| HFor ^e^ | 3.55 | 3.55 | [5] | 3.72 | 3.72 |
|  | *I = 0 mol L^-1^, T = 298.15 K* | |  | *I = 0 mol L^-1^, T = 298.15 K* | |
| HOx^- f^ | 4.27 | 4.27 | [5] | 4.27 | 4.27 |
| H_2_Ox | 1.27 | 5.54 |  | 1.27 | 5.54 |
| HMal^- g^ | 5.70 | 5.70 |  | 5.70 | 5.70 |
| H_2_Mal | 2.83 | 8.53 |  | 2.83 | 8.53 |
| HNO_2_ | 3.15 | 3.15 |  | 3.15 | 3.15 |
| NH_4_^+^ | *9.245* | 9.245 |  | 9.24 | 9.24 |
|  | *I = 0.1 mol L^-1^, NaCl, T = 298.15 K* | |  | *I = 0 mol L^-1^, T = 298.15 K* | |
| OH^-^ | -13.775 | -13.775 | [5] | -13.997 | -13.997 |

^a^ log*K* values refer to the general reaction: H_r−1_L^z−(r−1)^ + H^+^ ⇄ H_r_L^z−r^, with z = charge of the fully deprotonated ligand;
^b^ log*β* values refer to the general reaction: L^z−^ + rH^+^ ⇄ H_r_L^z−r^;
^c^ NEt_4_I: tetraethylammonium iodide;
^d^ Ac: acetate;
^e^ For: formate;
^f^ Ox: oxalate;
^g^ Mal: malonate

Table S3. Stepwise (log*K*) and overall (logβ) protonation constant of the species considered in the model.

| **Formation constants** | | | | | |
| --- | --- | --- | --- | --- | --- |
| **Species** | **log*K ^a^*** | **log*β ^b^*** | **Ref.** | **log*K*** | **log*β*** |
|  | **Original values** | |  | **Values estimated by the application of EDH equation** [1] | |
|  | *I = 0 mol L^-1^, T = 298.15 K* | |  | *I = 0 mol L^-1^, T = 298.15 K* | |
| [CaOH]^+^ | 1.3 |  | [5] | 1.3 | -12.69 |
| [MgOH]^+^ |  | -11.44 |  |  | -11.44 |
| [Mg_4_OH_4_]^4+^ |  | -39.71 |  |  | -39.71 |
| [CuOH]^+^ |  | -7.70 |  |  | -7.70 |
| Cu(OH)_2_ |  | -17.30 |  |  | -17.30 |
| [Cu(OH)_3_]^-^ |  | -27.80 |  |  | -27.80 |
| [Cu(OH)_4_]^2-^ |  | -39.60 |  |  | -39.60 |
| [Cu_2_(OH)_2_]^2+^ |  | -10.36 |  |  | -10.36 |
| [MnOH]^+^ |  | -10.59 |  |  | -10.59 |
| Mn(OH)_2_ |  | -22.20 |  |  | -22.20 |
| [Mn(OH)_3_]^-^ |  | -34.80 |  |  | -34.80 |
| [Mn(OH)_4_]^2-^ |  | -48.30 |  |  | -48.30 |
| [Mn_2_OH]^3+^ |  | -10.56 |  |  | -10.56 |
| [Mn_2_OH_3_]^+^ |  | -23.90 |  |  | -23.90 |
| [ZnOH]^+^ |  | -8.96 |  |  | -8.96 |
| Zn(OH)_2_ |  | -16.90 |  |  | -16.90 |
| [Zn(OH)_3_]^-^ |  | -28.40 |  |  | -28.40 |
| [Zn(OH)_4_]^2-^ |  | -41.20 |  |  | -41.20 |
| [Zn_2_OH]^3+^ |  | -9.00 |  |  | -9.00 |
| [Zn_2_(OH)_6_]^2-^ |  | -57.80 |  |  | -57.80 |
| [FeOH]^2+^ |  | -2.19 |  |  | -2.19 |
| [Fe(OH)_2_]^+^ |  | -5.67 |  |  | -5.67 |
| Fe(OH)_3_ |  | -12.92 |  |  | -12.92 |
| [Fe(OH)_4_]^-^ |  | -24.4 |  |  | -24.4 |
| [Fe_2_(OH)_2_]^4+^ |  | -2.95 |  |  | -2.95 |
| [Fe_3_(OH)_4_]^5+^ |  | -6.30 |  |  | -6.30 |
| [AlOH]^2+^ |  | -5.00 |  |  | -5.00 |
| [Al(OH)_2_]^+^ |  | -10.30 |  |  | -10.30 |
| [Al(OH)_3_] |  | -16.20 |  |  | -16.20 |
| [Al(OH)_4_]^-^ |  | -22.20 |  |  | -22.20 |
| [Al_2_(OH)_2_]^4+^ |  | -7.55 |  |  | -7.55 |
| [Al_3_(OH)_4_]^5+^ |  | -13.9 |  |  | -13.9 |
|  |  |  |  |  |  |
|  | *I = 0 mol L^-1^, KCl, T = 298.15 K* | |  | *I = 0 mol L^-1^, T = 298.15 K* | |
| NaCl | -0.30 | -0.30 | [6] | -0.30 | -0.30 |
| KCl | -0.27 | -0.27 |  | -0.27 | -0.27 |
| [CaCl]^+^ | 0.57 | 0.57 |  | 0.57 | 0.57 |
| [MgCl]^+^ | 0.40 | 0.40 |  | 0.40 | 0.40 |
|  | *I = 1.0 mol L^-1^, NaClO_4_, T = 298.15 K* | |  | *I = 0 mol L^-1^, T = 298.15 K* | |
| [MnCl]^+^ |  | -0.09 | [5] |  | 0.46 |
| [Mn(Cl)_2_] |  | -0.52 |  |  | 0.26 |
|  | *I = 0 mol L^-1^, NaClO_4_, T = 298.15 K* | |  | *I = 0 mol L^-1^, T = 298.15 K* | |
| [CuCl]^+^ | 0.83 | 0.83 | [5] | 0.83 | 0.83 |
| CuCl_2_ | -0.23 | 0.60 |  | -0.23 | 0.60 |
|  | *I = 0 mol L^-1^, NaCl, T = 298.15 K* | |  | *I = 0 mol L^-1^, T = 298.15 K* | |
| [ZnCl]^+^ | 0.43 | 0.43 | [5] | 0.43 | 0.43 |
| ZnCl_2_ | 0.18 | 0.61 |  | 0.18 | 0.61 |
| [ZnCl_3_]^-^ | -0.10 | 0.51 |  | -0.10 | 0.51 |
| [ZnCl_4_]^2-^ | -0.31 | 0.20 |  | -0.31 | 0.20 |
| [FeCl]^2+^ | 1.28 | 1.28 | [7] | 1.28 | 1.28 |
| [FeCl_2_]^+^ | 1.16 | 2.44 |  | 1.16 | 2.44 |
|  | *I = 0.16 mol L^-1^, NEt_4_I ^c^, T = 298.15 K* | |  | *I = 0 mol L^-1^, T = 298.15 K* | |
| NaH_2_PO_4_ | 0.09 | 18.69 | [3] | 1.18 | 19.78 |
| [NaHPO_4_]^-^ | 0.69 | 12.48 |  | 1.60 | 13.39 |
| [NaPO_4_]^2-^ | 0.88 | 0.88 |  | 1.43 | 1.43 |
| Na_2_HPO_4_ | 0.44 | 12.23 |  | 1.53 | 13.32 |
| [Na_2_PO_4_]^-^ | 1.68 | 1.68 |  | 2.59 | 2.59 |
|  | *I = 0.16 mol L^-1^, NEt_4_I, T = 298.15 K* | |  | *I = 0 mol L^-1^, T = 298.15 K* | |
| KH_2_PO_4_ | 0.07 | 18.67 | [3] | 1.16 | 19.76 |
| [KHPO_4_]^-^ | 0.50 | 12.29 |  | 1.41 | 13.20 |
| [KPO_4_]^2-^ | 0.81 | 0.81 |  | 1.36 | 1.36 |
| K_2_HPO_4_ | 0.56 | 12.35 |  | 1.65 | 13.44 |
| [K_2_PO_4_]^-^ | 1.28 | 1.28 |  | 2.19 | 2.19 |
|  | *I = 0.15 mol L^-1^, KCl, T = 298.15 K* | |  | *I = 0 mol L^-1^, T = 298.15 K* | |
| [CaH_2_PO_4_]^+^ | 1.41 |  | [8] | 1.86 | 21.41 |
| CaHPO_4_ | 2.74 |  |  | 3.65 | 16.00 |
| [CaPO_4_]^-^ | 6.46 |  |  | 7.83 | 7.83 |
|  | *I = 0.2 mol L^-1^, NEt_4_I, T = 298.15 K* | |  | *I = 0 mol L^-1^, T = 298.15 K* | |
| MgHPO_4_ | 2.41 |  | [9] | 2.63 | 14.98 |
|  | *I = 0.1 mol L^-1^, NaClO_4_, T = 298.15 K* | |  | *I = 0 mol L^-1^, T = 298.15 K* | |
| CuHPO_4_ |  | 14.93 | [5] |  | 16.52 |
|  | *I = 0.2 mol L^-1^, NEt_4_I, T = 298.15 K* | |  | *I = 0 mol L^-1^, T = 298.15 K* | |
| MnHPO_4_ | 2.58 |  | [5] | 3.89 | 16.24 |
|  | *I = 0.1 mol L^-1^, NaNO_3_, T = 298.15 K* | |  | *I = 0 mol L^-1^, T = 298.15 K* | |
| ZnHPO_4_ | 2.4 |  | [5] | 3.2 | 15.6 |
|  | *I = 0.1 mol L^-1^, NaClO_4_, T = 298.15 K* | |  | *I = 0 mol L^-1^, T = 298.15 K* | |
| [FeH_2_PO_4_]^2+^ | 3.47 |  | [9] | 4.69 | 24.24 |
| [FeHPO_4_]^+^ | 8.95 |  | [5] | 10.17 | 22.52 |
| [Fe_2_HPO_4_]^4+^ | 6.17 |  |  | 6.77 | 29.29 |
|  | *I = 0.2 mol L^-1^, KCl, T = 298.15 K* | |  | *I = 0 mol L^-1^, T = 298.15 K* | |
| [AlH_2_PO_4_]^2+^ |  | 19.65 | [5] |  | 21.83 |
| [AlHPO_4_]^+^ |  | 17.60 |  |  | 19.79 |
| [Al_2_PO_4_]^3+^ |  | 17.42 |  |  | 19.59 |
|  | *I = 0 mol L^-1^, NEt_4_I, T = 298.15 K* | |  | *I = 0 mol L^-1^, T = 298.15 K* | |
| [NaSO_4_]^-^ | 0.65 | 0.65 | [10] | 0.65 | 0.65 |
| [KSO_4_]^-^ | 0.75 | 0.75 |  | 0.75 | 0.75 |
| CaSO_4_ | 2.43 | 2.43 |  | 2.43 | 2.43 |
| MgSO_4_ | 2.23 | 2.23 |  | 2.23 | 2.23 |
| CuSO_4_ | 2.26 | 2.26 |  | 2.26 | 2.26 |
| MnSO_4_ | 2.86 | 2.86 |  | 2.86 | 2.86 |
| ZnSO_4_ | 2.49 | 2.49 |  | 2.49 | 2.49 |
| [FeSO_4_]^+^ | 4.27 | 4.27 | [7] | 4.27 | 4.27 |
| [Fe(SO_4_)_2_]^-^ |  | 6.11 |  |  | 6.11 |
|  | *I = 0 mol L^-1^, R_4_NX ^d^, T = 298.15 K* | |  | *I = 0 mol L^-1^, T = 298.15 K* | |
| [AlSO_4_]^+^ |  | 3.84 | [5] |  | 3.84 |
| [Al(SO_4_)_2_]^-^ |  | 5.58 |  |  | 5.58 |
|  | *I = 0 mol L^-1^, T = 298.15 K* | |  | *I = 0 mol L^-1^, T = 298.15 K* | |
| [NaNO_2_] |  | -0.42 | [5] |  | -0.42 |
| [KNO_2_] |  | -0.11 |  |  | -0.11 |
|  | *I = 1.0 mol L^-1^, NaClO_4_, T = 298.15 K* | |  | *I = 0 mol L^-1^, T = 298.15 K* | |
| [MnNO_2_]^+^ |  | 0.45 | [5] |  | 1.00 |
| [CuNO_2_]^+^ |  | 1.34 |  |  | 1.89 |
| [Cu(NO_2_)_2_] |  | 1.68 |  |  | 2.48 |
| [ZnNO_2_]^+^ |  | 0.37 |  |  | 0.92 |
| [Zn(NO_2_)_2_] |  | 0.49 |  |  | 1.27 |
| [FeNO_2_]^2+^ |  | 2.59 |  |  | 3.77 |
| [Fe(NO_2_)_2_]^+^ |  | 3.7 |  |  | 5.1 |
| [Fe(NO_2_)_3_] |  | 5.45 |  |  | 7.10 |

|  | *I = 0 mol L^-1^, T = 298.15 K* | |  | *I = 0 mol L^-1^, T = 298.15 K* | |
| --- | --- | --- | --- | --- | --- |
| [NaNO_3_] |  | -0.57 | [5] |  | -0.57 |
| [KNO_3_] |  | -0.22 |  |  | -0.22 |
|  | *I = 0.5 mol L^-1^, NaClO_4_, T = 298.15 K* | |  | *I = 0 mol L^-1^, T = 298.15 K* | |
| [CaNO_3_]^+^ |  | 0.06 | [5] |  | 0.60 |
| Ca(NO_3_)_2_ |  | -0.30 |  |  | 0.50 |
| Mn(NO_3_)_2_ |  | -0.30 |  |  | 0.50 |
|  | *I = 2.0 mol L^-1^, T = 298.15 K* | |  | *I = 0 mol L^-1^, T = 298.15 K* | |
| [CuNO_3_]^+^ |  | -0.06 | [9] |  | 0.49 |
| Cu(NO_3_)_2_ |  | -0.6 |  |  | 0.14 |
|  | *I = 0 mol L^-1^, T = 298.15 K* | |  | *I = 0 mol L^-1^, T = 298.15 K* | |
| [ZnNO_3_]^+^ |  | 0.40 | [9] |  | 0.40 |
| Zn(NO_3_)_2_ |  | -0.30 |  |  | -0.30 |
| [Fe(NO_3_)]^2+^ |  | 1.00 |  |  | 1.00 |
|  | *I = 0.0 mol L^-1^, NEt_4_I, T = 298.15 K* | |  | *I = 0 mol L^-1^, T = 298.15 K* | |
| [Na(Ac)] ^e^ | -0.11 | -0.11 | [4] | -0.11 | -0.11 |
| [K(Ac)] | -0.27 | -0.27 |  | -0.27 | -0.27 |
| [Ca(Ac)]^+^ | 1.12 | 1.12 | [11] | 1.12 | 1.12 |
| [Mg(Ac)]^+^ | 0.91 | 0.91 | [5] | 0.91 | 0.91 |
|  | *I = 0.1 mol L^-1^, NaClO_4_, T = 298.15 K* | |  | *I = 0 mol L^-1^, T = 298.15 K* | |
| [Cu(Ac)]^+^ | 1.78 | 1.78 | [5] | 2.18 | 2.18 |
| [Cu(Ac)_2_] | 1.02 | 2.80 |  | 1.22 | 3.40 |
|  | *I = 0.1 mol L^-1^, KCl, T = 298.15 K* | |  | *I = 0.1 mol L^-1^, T = 298.15 K* | |
| [Mn(Ac)]^+^ | 0.80 | 0.80 | [5] | 1.20 | 1.20 |
|  | *I = 0.1 mol L^-1^, KNO_3_, T = 298.15 K* | |  | *I = 0 mol L^-1^, T = 298.15 K* | |
| [Zn(Ac)]^+^ | 1.11 | 1.11 | [5] | 1.51 | 1.51 |
|  | *I = 0 mol L^-1^, NaClO_4_, T = 298.15 K* | |  | *I = 0 mol L^-1^, T = 298.15 K* | |
| [Fe(Ac)]^2+^ |  | 4.07 | [5] |  | 4.07 |
| [Fe(Ac)_2_]^+^ |  | 8.81 |  |  | 8.81 |
| [Fe_3_(OH)_3_(Ac)_3_]^3+^ |  | 11.37 |  |  | 11.37 |
|  | *I = 0.1 mol L^-1^, NaCl, T = 298.15 K* | |  | *I = 0 mol L^-1^, T = 298.15 K* | |
| [AlAc]^2+^ |  | 2.02 | [5] |  | 2.83 |
| [Al(Ac)_2_]^+^ |  | 3.50 |  |  | 4.51 |
|  | *I = 0.03 mol L^-1^, NaCl, T = 298.15 K* | |  | *I = 0 mol L^-1^, T = 298.15 K* | |
| [Ca(For)]^+^ ^f^ | 0.75 | 0.75 | [5] | 1.01 | 1.01 |
| [Mg(For)]^+^ | 0.75 | 0.75 |  | 1.01 | 1.01 |
|  | *I = 0.1 mol L^-1^, NaNO_3_, T = 298.15 K* | |  | *I = 0 mol L^-1^, T = 298.15 K* | |
| [Cu(For)]^+^ | 1.58 | 1.58 | [5] | 1.98 | 1.98 |

|  | *I = 1.0 mol L^-1^, T = 298.15 K* | |  | *I* = 0 mol L^-1^, *T = 298.15 K* | |
| --- | --- | --- | --- | --- | --- |
| [MnFor]^+^ |  | 0.80 | [5] |  | 1.35 |
|  | *I = 0.1 mol L^-1^, KNO_3_, T = 298.15 K* | |  | *I = 0 mol L^-1^, T = 298.15 K* | |
| [Zn(For)]^+^ | 1.07 | 1.07 | [5] | 1.47 | 1.47 |
|  | *I = 0 mol L^-1^, T = 298.15 K* | |  | *I = 0 mol L^-1^, T = 298.15 K* | |
| [Fe(For)]^2+^ | 3.1 | 3.1 | [12] | 3.1 | 3.1 |
|  | *I = 1.0 mol L^-1^, NaNO_3_, T = 298.15 K* | |  | *I = 0 mol L^-1^, T = 298.15 K* | |
| [AlFor]^2+^ |  | 1.30 | [5] |  | 2.48 |
| [Al(For)_2_]^+^ |  | 2.02 |  |  | 3.43 |
|  | *I = 0.25 mol L^-1^, R_4_NX, T = 298.15 K* | |  | *I = 0 mol L^-1^, T = 298.15 K* | |
| [NaMal]^- g^ |  | 0.57 | [5] |  | 0.94 |
| [NaHMal] |  | 5.15 |  |  | 5.69 |
| [KMal]^-^ |  | 0.68 |  |  | 1.05 |
|  | *I = 0.1 mol L^-1^, NaCl, T = 298.15 K* | |  | *I = 0 mol L^-1^, T = 298.15 K* | |
| CaMal |  | 1.75 | [5] |  | 2.56 |
| [CaHMal]^+^ |  | 6.14 |  |  | 6.94 |
|  | *I = 0.1 mol L^-1^, NaClO_4_, T = 298.15 K* | |  | *I = 0 mol L^-1^, T = 298.15 K* | |
| MgMal |  | 2.11 | [5] |  | 2.92 |
| [MgHMal]^+^ |  | 6.24 |  |  | 7.04 |
|  | *I = 0.16 mol L^-1^, T = 298.15 K* | |  | *I = 0 mol L^-1^, T = 298.15 K* | |
| MnMal |  | 2.30 | [5] |  | 3.22 |
|  | *I = 0.1 mol L^-1^, KNO_3_, T = 298.15 K* | |  | *I = 0 mol L^-1^, T = 298.15 K* | |
| [CuMal] |  | 4.97 | [5] |  | 5.78 |
| [Cu(Mal)_2_]^2-^ |  | 7.77 |  |  | 8.71 |
|  | *I = 1.0 mol L^-1^, NaClO_4_, T = 298.15 K* | |  | *I = 0 mol L^-1^, T = 298.15 K* | |
| [CuHMal]^+^ | 1.90 | 6.97 | [5] | 3.00 | 8.07 |
| CuH_2_(Mal)_2_ | 2.66 | 12.8 |  | 3.91 | 14.68 |
|  | *I = 0.1 mol L^-1^, KNO_3_, T = 298.15 K* | |  | *I = 0 mol L^-1^, T = 298.15 K* | |
| [CuMalOx]^2- h^ |  | 8.54 | [5] |  | 9.55 |
|  | *I = 1.0 mol L^-1^, NaClO_4_, T = 298.15 K* | |  | *I = 0 mol L^-1^, T = 298.15 K* | |
| ZnMal |  | 2.47 | [5] |  | 3.65 |
| [Zn(Mal)_2_]^2-^ |  | 3.80 |  |  | 5.21 |
| [ZnHMal]^+^ | 0.59 | 5.66 |  | 1.69 | 6.76 |
|  | *I = 0.5 mol L^-1^, NaNO_3_, T = 298.15 K* | |  | *I = 0 mol L^-1^, T = 298.15 K* | |
| [FeMal]^+^ |  | 7.52 | [5] |  | 9.24 |
| [Fe(Mal)_2_]^-^ |  | 13.29 |  |  | 15.55 |
|  | *I = 1.0 mol L^-1^, NaClO_4_, T = 298.15 K* | |  | *I = 0 mol L^-1^, T = 298.15 K* | |
| [FeHMal]^2+^ | 2.80 | 7.87 | [5] | 4.13 | 9.20 |

|  | *I = 0 mol L^-1^, NaCl, T = 298.15 K* | |  | *I = 0 mol L^-1^, T = 298.15 K* | |
| --- | --- | --- | --- | --- | --- |
| [AlMal]^+^ |  | 7.49 | [5] |  | 7.49 |
| [Al(Mal)_2_]^-^ |  | 12.62 |  |  | 12.62 |
|  | *I = 1.0 mol L^-1^, NaClO_4_, T = 298.15 K* | |  | *I = 0 mol L^-1^, T = 298.15 K* | |
| [AlHMal]^2+^ | 3.58 | 8.65 | [5] | 4.99 | 10.38 |
| [AlH_2_(Mal)_2_]^+^ | 5.99 | 16.13 |  | 8.50 | 18.64 |
| AlH_3_(Mal)_3_ | 8.58 | 23.79 |  | 11.87 | 27.04 |
|  | *I = 0 mol L^-1^, R_4_NX, T = 298.15 K* | |  | *I = 0 mol L^-1^, T = 298.15 K* | |
| [NaOx]^-^ |  | 0.82 | [5] |  | 0.82 |
|  | *I = 0.04 mol L^-1^, NaCl, T = 298.15 K* | |  | *I = 0 mol L^-1^, T = 298.15 K* | |
| [KOx]^-^ |  | 0.43 | [5] |  | 0.73 |
| KHOx |  | 3.60 |  |  | 4.05 |
|  | *I = 1.0 mol L^-1^, NaClO_4_, T = 298.15 K* | |  | *I = 0 mol L^-1^, T = 298.15 K* | |
| CaOx |  | 1.66 | [5] |  | 2.84 |
| [Ca(Ox)_2_]^2-^ |  | 2.69 |  |  | 4.10 |
|  | *I = 0.1 mol L^-1^, T = 298.15 K* | |  | *I = 0 mol L^-1^, T = 298.15 K* | |
| [CaHOx]^+^ | 1.38 | 5.20 | [5] | 2.18 | 6.00 |
| [Ca(HOx)_2_] | 1.85 | 9.49 |  | 3.25 | 10.89 |
|  | *I = 0.15 mol L^-1^, NaCl, T = 298.15 K* | |  | *I = 0 mol L^-1^, T = 298.15 K* | |
| MgOx |  | 2.18 | [5] |  | 3.09 |
| [Mg(Ox)2]^2-^ |  | 4.24 |  |  | 5.22 |
|  | *I = 0.1 mol L^-1^, NaClO_4_, T = 298.15 K* | |  | *I = 0 mol L^-1^, T = 298.15 K* | |
| MnOx |  | 3.15 | [5] |  | 3.96 |
| [Mn(Ox)_2_]^2-^ |  | 4.41 |  |  | 5.42 |
|  | *I = 0.1 mol L^-1^, KNO_3_, T = 298.15 K* | |  | *I = 0 mol L^-1^, T = 298.15 K* | |
| CuOx |  | 4.28 | [5] |  | 5.68 |
| [Cu(Ox)_2_]^2-^ |  | 8.25 |  |  | 9.26 |
|  | *I = 0.1 mol L^-1^, T = 298.15 K* | |  | *I = 0 mol L^-1^, T = 298.15 K* | |
| [CuHOx]^+^ | 2.49 | 6.31 | [9] | 3.29 | 7.11 |
|  | *I = 1.0 mol L^-1^, NaClO_4_, T = 298.15 K* | |  | *I = 0 mol L^-1^, T = 298.15 K* | |
| ZnOx |  | 3.60 | [5] |  | 4.78 |
| [Zn(Ox)_2_]^2-^ |  | 6.15 |  |  | 6.51 |
|  | *I = 0.1 mol L^-1^, T = 298.15 K* | |  | *I = 0 mol L^-1^, T = 298.15 K* | |
| [ZnHOx]^+^ | 1.72 | 5.54 | [9] | 2.52 | 6.34 |
| Zn(HOx)_2_ | 3.12 | 10.76 |  | 4.52 | 12.16 |
|  | *I = 0.5 mol L^-1^, NaClO_4_, T = 298.15 K* | |  | *I = 0 mol L^-1^, T = 298.15 K* | |
| [FeOx]^+^ |  | 7.53 | [5] |  | 9.25 |
| [Fe(Ox)_2_]^-^ |  | 13.64 |  |  | 15.90 |
| [Fe(Ox)_3_]^3-^ |  | 18.49 |  |  | 21.00 |

|  | *I = 0.5 mol L^-1^, T = 298.15 K* | |  | *I = 0 mol L^-1^, T = 298.15 K* | |
| --- | --- | --- | --- | --- | --- |
| [FeHOx]^2+^ | 4.35 | 7.99 | [5] | 5.73 | 9.37 |
|  | *I = 0 mol L^-1^, T = 298.15 K* | |  | *I = 0 mol L^-1^, T = 298.15 K* | |
| [AlOx]^+^ |  | 7.18 | [5] |  | 7.18 |
| [Al(Ox)_2_]^-^ |  | 13.49 |  |  | 13.49 |
| [Al(Ox)_3_]^3-^ |  | 17.53 |  |  | 17.53 |

^a^ log*K* values refer to the general reaction: pM^n+^ + qH_r_L^z-r^ ⮀ [M_p_L_q_H_r_]^np+qr-qz^, with z = charge of the fully deprotonated ligand; for hydrolytic species the reaction is: pM^n+^ + qOH^-^ ⮀ [M_p_(OH)_q_]^np-q^

^b^ logβ values refer to the general reaction: pM^n+^ + qL^z-^ + rH^+^ ⮀ [M_p_L_q_H_r_]^np+qr-qz^; for hydrolytic species the reaction is: pM^n+^ + qH_2_O ⮀ [M_p_(OH)_q_]^np-q^ + qH^+^

^c^ NEt_4_I: tetraethylammonium iodide;

^d^ R_4_NX: tetraalkylammonium salts;

^e^ Ac: acetate;

^f^ For: formate;

^g^ Mal: malonate;
^h^ Ox: oxalate.

Table S4. Solubility constant (logK_s_) of the major solid species considered in the model.

| **Solubility constant** | | | |
| --- | --- | --- | --- |
| **Species** | **log*Ks*** | **Ref.** | **log*Ks*** |
|  | **Original values** |  | **Values estimated by the application of EDH equation** [1] |
|  | *I* = 0 mol L^-1^, *T = 298.15 K* |  | *I* = 0 mol L^-1^, *T = 298.15 K* |
| Mn(OH)_2_ | 15.20 | [5] | 15.20 |
| Cu(OH)_2_ | 7.60 |  | 7.60 |
| Zn(OH)_2_ | 12.40 |  | 12.40 |
| Fe(OH)_3_ | 3.79 |  | 3.79 |
| Al(OH)_3_ | 8.11 |  | 8.11 |
| NaCl | 1.55 |  | 1.55 |
| KCl | 0.93 |  | 0.93 |
| CaSO_4_ | -4.26 |  | -4.26 |

Table S5. Concentration (mol L^-1^) of the components of the soluble fraction of PM_10_ samples collected at Ny-Ålesund during the spring-summer campaign of 2012. Values below the instrumental limit of detection have been recalculated by PCA (italic) or are replaced by LOD value (underlined).

| Sample | Date | Volume (m^3^) | f ^a^ | Al | Fe | Cu | Mn | Zn | Na | K | Mg | Ca | NH_4_^+^ | Acetate | Formate | Cl^-^ | NO_2_^-^ | NO_3_^-^ | SO_4_^2-^ | Malonate | Oxalate | PO_4_^3-^ |
| --- | --- | --- | --- | --- | --- | --- | --- | --- | --- | --- | --- | --- | --- | --- | --- | --- | --- | --- | --- | --- | --- | --- |
| 8 | 21/04/2012 | 1090.68 | 0.96 | 7.62E-07 | 2.64E-07 | 3.47E-08 | 5.53E-08 | 1.06E-07 | 1.64E-04 | 5.95E-06 | 2.54E-05 | 1.44E-05 | 7.59E-05 | *5.37E-07* | 3.05E-06 | 1.40E-04 | 3.71E-07 | 2.36E-05 | 8.31E-05 | *6.28E-07* | 2.49E-06 | 1.13E-05 |
| 12 | 07/05/2012 | 1059.76 | 0.99 | 1.60E-06 | 8.02E-07 | 1.40E-08 | 8.59E-08 | 2.05E-07 | 1.32E-04 | 4.93E-06 | 1.90E-05 | 1.47E-05 | 1.10E-04 | 4.44E-07 | 2.08E-05 | 4.03E-05 | 8.32E-07 | 2.89E-05 | 1.32E-04 | 5.70E-07 | 3.10E-06 | 1.13E-05 |
| 13 | 11/05/2012 | 1085.68 | 0.97 | 1.32E-06 | 7.86E-07 | 1.78E-08 | 6.96E-08 | 1.08E-07 | 1.04E-04 | 5.36E-06 | 1.84E-05 | 1.22E-05 | 5.31E-05 | *6.54E-07* | 2.00E-05 | 2.83E-05 | *7.11E-07* | 7.27E-06 | 1.52E-04 | *7.93E-07* | *3.59E-06* | 1.13E-05 |
| 14 | 15/05/2012 | 1087.72 | 0.97 | 1.70E-06 | 7.76E-07 | 1.93E-08 | 1.61E-07 | 2.43E-07 | 1.73E-04 | 7.32E-06 | 2.39E-05 | 1.95E-05 | 1.38E-04 | 9.41E-07 | 1.63E-05 | 7.18E-05 | 2.29E-07 | 2.89E-05 | 1.10E-04 | 8.02E-07 | 4.45E-06 | 1.13E-05 |
| 15 | 19/05/2012 | 1051.59 | 1.00 | 1.22E-06 | 3.57E-07 | 1.58E-08 | 9.47E-08 | 1.19E-07 | 2.18E-04 | 7.74E-06 | 3.34E-05 | 1.92E-05 | 7.67E-05 | *6.40E-07* | 9.31E-06 | 1.59E-04 | 1.06E-06 | 2.84E-05 | 1.02E-04 | *7.55E-07* | 3.38E-06 | 1.13E-05 |
| 16 | 23/05/2012 | 997.82 | 1.05 | 9.82E-07 | 2.93E-07 | *1.33E-08* | 9.58E-08 | 1.51E-07 | 1.92E-04 | 5.14E-06 | 2.42E-05 | 1.61E-05 | 4.65E-05 | 6.93E-07 | 1.23E-05 | 1.02E-04 | 8.84E-07 | 2.49E-05 | 7.93E-05 | 3.36E-07 | 3.15E-06 | 1.13E-05 |
| 17 | 27/05/2012 | 1091.42 | 0.96 | 5.14E-07 | 3.17E-07 | 1.81E-08 | 4.15E-08 | 1.33E-07 | 6.80E-04 | 1.65E-05 | 8.89E-05 | 2.38E-05 | 2.38E-05 | *5.88E-07* | 5.86E-06 | 3.83E-04 | 3.81E-06 | 9.64E-06 | 2.62E-04 | *6.02E-07* | 1.79E-06 | 1.13E-05 |
| 18 | 31/05/2012 | 1040.93 | 1.01 | 1.62E-06 | 8.85E-07 | 2.01E-08 | 5.48E-08 | 1.88E-07 | 1.81E-04 | 4.62E-06 | 2.33E-05 | 1.17E-05 | 1.41E-04 | 4.52E-07 | 5.58E-06 | 3.25E-05 | 6.55E-08 | 9.39E-06 | 1.83E-04 | 9.02E-07 | 2.71E-06 | 1.13E-05 |
| 19 | 04/06/2012 | 1054.97 | 1.00 | 6.44E-07 | 2.69E-07 | 9.07E-09 | 4.50E-08 | 9.09E-08 | 2.27E-04 | 6.91E-06 | 3.19E-05 | 1.36E-05 | 3.48E-05 | *5.85E-07* | 9.86E-06 | 1.13E-04 | 1.24E-06 | 4.04E-05 | 9.99E-05 | 4.52E-07 | 3.42E-06 | 1.13E-05 |
| 20 | 08/06/2012 | 1092.18 | 0.96 | 4.32E-07 | 1.80E-07 | 6.78E-09 | 2.81E-08 | 9.41E-08 | 4.04E-04 | 8.12E-06 | 4.78E-05 | 1.46E-05 | 3.11E-05 | 1.04E-06 | 2.07E-05 | 1.90E-04 | 1.45E-07 | 9.88E-05 | 1.05E-04 | 1.35E-06 | 6.32E-06 | 1.13E-05 |
| 21 | 12/06/2012 | 998.35 | 1.05 | 4.72E-07 | 1.98E-07 | 6.34E-09 | 5.49E-08 | 1.78E-07 | 7.67E-05 | 2.80E-06 | 1.54E-05 | 1.44E-05 | 6.62E-05 | *5.84E-07* | 9.00E-06 | 1.86E-05 | 1.42E-06 | 2.13E-05 | 7.41E-05 | *7.36E-07* | 3.90E-06 | 1.13E-05 |
| 22 | 16/06/2012 | 1016.03 | 1.03 | 4.77E-07 | 1.86E-07 | 6.65E-09 | 2.38E-08 | 7.33E-08 | 2.50E-04 | 5.15E-06 | 2.99E-05 | 1.06E-05 | 3.95E-05 | 8.99E-07 | 3.94E-05 | 8.99E-05 | 2.45E-07 | 2.27E-05 | 9.68E-05 | 1.52E-06 | 5.92E-06 | 1.13E-05 |
| 23 | 20/06/2012 | 1013.04 | 1.04 | 2.12E-07 | 9.91E-08 | 6.17E-09 | 1.42E-08 | 5.47E-08 | 4.57E-04 | 1.16E-05 | 5.96E-05 | 1.66E-05 | 7.04E-06 | *6.62E-07* | 1.85E-05 | 4.47E-04 | *1.16E-06* | 1.74E-05 | 7.25E-05 | *8.35E-07* | 3.29E-06 | 1.13E-05 |
| 24 | 24/06/2012 | 1011.73 | 1.04 | 1.91E-07 | 1.04E-09 | 4.37E-09 | 1.67E-09 | 1.05E-07 | 6.17E-06 | *1.67E-06* | 1.11E-06 | 3.72E-06 | 7.59E-07 | 1.36E-07 | 3.43E-07 | 1.44E-05 | 1.56E-07 | *2.24E-05* | 8.34E-07 | *5.36E-07* | *1.34E-06* | 1.13E-05 |
| 25 | 28/06/2012 | 1026.34 | 1.02 | 8.66E-08 | 2.04E-08 | 6.63E-09 | 7.14E-09 | 2.05E-07 | 8.11E-05 | 2.35E-06 | 1.18E-05 | 6.84E-06 | 1.58E-05 | *4.06E-07* | 5.75E-06 | 5.29E-05 | *4.12E-07* | 1.47E-05 | 3.04E-05 | *5.28E-07* | 1.10E-06 | 1.13E-05 |
| 26 | 02/07/2012 | 1003.29 | 1.05 | 5.06E-07 | 1.60E-07 | 1.53E-08 | 2.73E-08 | 1.05E-07 | 3.13E-04 | 7.39E-06 | 3.84E-05 | 1.38E-05 | 7.67E-06 | 1.13E-06 | 1.74E-05 | 2.13E-04 | *5.30E-07* | 5.01E-05 | 5.98E-05 | 1.40E-06 | 5.75E-06 | 1.13E-05 |
| 27 | 06/07/2012 | 1037.11 | 1.01 | 8.35E-08 | 2.84E-08 | 2.06E-09 | 2.69E-08 | 6.26E-08 | 2.66E-04 | 6.26E-06 | 3.69E-05 | 1.62E-05 | *1.02E-05* | *4.45E-07* | 6.78E-06 | 2.77E-04 | *8.81E-07* | 4.99E-06 | 3.65E-05 | *5.52E-07* | 5.00E-07 | 1.13E-05 |
| 28 | 10/07/2012 | 1026.30 | 1.02 | 1.63E-07 | 1.09E-07 | 2.25E-09 | 5.70E-08 | 6.99E-08 | 2.06E-04 | 4.04E-06 | 3.08E-05 | 1.98E-05 | 2.52E-06 | 3.51E-07 | 2.18E-06 | 1.89E-04 | *4.47E-07* | 1.41E-05 | 2.86E-05 | 4.57E-07 | 2.79E-06 | 1.13E-05 |
| 29 | 14/07/2012 | 1029.54 | 1.02 | 1.87E-07 | 1.09E-07 | 4.00E-09 | 1.98E-08 | 8.46E-08 | 1.34E-04 | 3.71E-06 | 2.04E-05 | 1.21E-05 | 5.49E-06 | *5.19E-07* | 2.61E-06 | 1.01E-04 | 1.92E-06 | 9.74E-06 | 3.48E-05 | *6.80E-07* | 3.58E-06 | 1.13E-05 |
| 33 | 30/07/2012 | 1098.77 | 0.96 | 5.96E-07 | 9.91E-08 | 1.04E-08 | 1.72E-08 | 1.01E-07 | 2.94E-04 | 6.06E-06 | 3.55E-05 | 1.28E-05 | 8.60E-06 | 4.28E-07 | 4.24E-06 | 2.88E-04 | 6.17E-08 | 2.21E-05 | 3.42E-05 | 7.94E-07 | 2.73E-06 | 1.13E-05 |
| 34 | 03/08/2012 | 1075.06 | 0.98 | 4.58E-08 | *1.35E-08* | 3.00E-09 | 1.31E-09 | 3.34E-08 | 1.28E-04 | 3.40E-06 | 1.81E-05 | 7.38E-06 | 1.51E-06 | *4.45E-07* | 7.98E-07 | 1.57E-04 | *3.39E-07* | 3.23E-06 | 1.35E-05 | *5.99E-07* | *1.82E-06* | 1.13E-05 |
| 35 | 07/08/2012 | 1074.20 | 0.98 | 3.62E-07 | 1.13E-07 | 4.47E-09 | 1.63E-08 | 7.79E-08 | 1.61E-04 | 3.99E-06 | 1.99E-05 | 8.97E-06 | 2.06E-06 | 3.35E-07 | 5.12E-06 | 1.61E-04 | 6.36E-08 | 3.02E-06 | 2.35E-05 | 3.12E-07 | 1.57E-06 | 1.13E-05 |
| 36 | 11/08/2012 | 919.08 | 1.14 | 7.94E-08 | 7.15E-09 | 3.46E-09 | 3.35E-09 | 5.77E-08 | 1.16E-04 | 4.19E-06 | 1.65E-05 | 8.67E-06 | 4.40E-06 | *4.74E-07* | 1.43E-05 | 1.07E-04 | *7.78E-07* | 7.07E-05 | 2.50E-05 | *6.42E-07* | 1.15E-06 | 3.30E-05 |
| 37 | 15/08/2012 | 1088.64 | 0.96 | 1.30E-07 | 3.16E-08 | 2.99E-09 | 9.87E-09 | 3.95E-08 | 9.21E-05 | 1.86E-06 | 1.20E-05 | 6.71E-06 | 5.45E-06 | 7.37E-07 | 3.40E-06 | 7.93E-05 | 3.11E-07 | 5.25E-06 | 1.79E-05 | 2.46E-07 | 8.67E-07 | 1.13E-05 |

| Sample | Date | Volume (m^3^) | f ^a^ | Al | Fe | Cu | Mn | Zn | Na | K | Mg | Ca | NH_4_^+^ | Acetate | Formate | Cl^-^ | NO_2_^-^ | NO_3_^-^ | SO_4_^2-^ | Malonate | Oxalate | PO_4_^3-^ |
| --- | --- | --- | --- | --- | --- | --- | --- | --- | --- | --- | --- | --- | --- | --- | --- | --- | --- | --- | --- | --- | --- | --- |
| 38 | 19/08/2012 | 1057.32 | 0.99 | 6.75E-08 | 2.23E-08 | 2.10E-09 | 2.52E-08 | 5.18E-08 | 2.60E-04 | 6.64E-06 | 3.60E-05 | 1.48E-05 | *6.18E-06* | *4.13E-07* | 1.18E-06 | 3.00E-04 | 2.48E-07 | 3.93E-06 | 2.34E-05 | *5.15E-07* | 2.50E-07 | 1.13E-05 |
| 39 | 23/08/2012 | 1074.06 | 0.98 | 5.20E-08 | 3.21E-08 | 1.86E-09 | 2.11E-08 | 4.78E-08 | 1.85E-04 | 3.75E-06 | 2.32E-05 | 9.67E-06 | 4.87E-06 | 1.29E-07 | 3.75E-06 | 1.97E-04 | 6.36E-08 | 2.42E-06 | 2.54E-05 | *4.70E-07* | 5.17E-07 | 1.13E-05 |
| 40 | 27/08/2012 | 1073.32 | 0.98 | 3.71E-07 | 5.07E-08 | 1.03E-08 | 9.58E-09 | 5.01E-08 | 1.90E-04 | 6.71E-06 | 2.61E-05 | 1.02E-05 | 1.04E-05 | *7.61E-07* | 5.40E-05 | 1.95E-04 | 1.88E-06 | 2.64E-04 | 3.72E-05 | *1.03E-06* | 2.28E-06 | 1.26E-04 |
| 41 | 31/08/2012 | 1082.66 | 0.97 | 1.25E-07 | 4.75E-08 | 6.93E-09 | 1.43E-08 | 1.08E-07 | 5.54E-04 | 1.27E-05 | 6.48E-05 | 1.63E-05 | 1.89E-05 | 3.32E-07 | 3.23E-06 | 5.93E-04 | 6.27E-08 | 1.62E-05 | 5.33E-05 | 2.46E-07 | 1.78E-06 | 1.13E-05 |
| 42 | 04/09/2012 | 1062.70 | 0.99 | 1.90E-08 | 3.70E-09 | 1.33E-09 | 3.46E-09 | 5.07E-08 | 3.23E-04 | 8.85E-06 | 4.01E-05 | 1.31E-05 | 3.78E-08 | *5.05E-07* | 2.08E-06 | 3.73E-04 | *8.15E-07* | 1.22E-05 | 2.62E-05 | *6.46E-07* | *2.04E-06* | 1.13E-05 |

^a^ multiplicative correction factor used to correct the concentrations in order to be all referred to the average volume of sampled air: $f_{i}=\bar{V}/{V_{i}}$, $V_{i}$ = volume of air collected, $\bar{V}$ = average volume of air collected.

Table S6. Uncertainties (standard deviation, mol L^-1^) of the concentration of the components of the soluble fraction of PM_10_ samples collected at Ny-Ålesund during the spring-summer campaign of 2012.

| Sample | Date | Volume (m^3^) | f ^a^ | Al | Fe | Cu | Mn | Zn | Na | K | Mg | Ca | NH_4_^+^ | Acetate | Formate | Cl^-^ | NO_2_^-^ | NO_3_^-^ | SO_4_^2-^ | Malonate | Oxalate | PO_4_^3-^ |
| --- | --- | --- | --- | --- | --- | --- | --- | --- | --- | --- | --- | --- | --- | --- | --- | --- | --- | --- | --- | --- | --- | --- |
| 8 | 21/04/2012 | 1090.68 | 0.96 | 3.50E-08 | 8.08E-08 | 9.98E-10 | 5.15E-09 | 1.38E-08 | 1.70E-05 | 2.97E-06 | 1.29E-06 | 8.57E-07 | 4.85E-06 | n.a. | n.a. | 1.01E-06 | 2.57E-06 | 2.22E-07 | n.a. | 3.30E-06 | 1.58E-06 | n.a. |
| 12 | 07/05/2012 | 1059.76 | 0.99 | 7.23E-08 | 9.45E-08 | 3.60E-09 | 8.15E-09 | 1.22E-08 | 2.87E-06 | 1.49E-06 | 1.13E-06 | 5.76E-07 | 2.40E-05 | n.a. | 2.06E-07 | 8.36E-07 | 1.29E-06 | 1.89E-07 | n.a. | 1.57E-06 | 6.74E-06 | 1.46E-07 |
| 13 | 11/05/2012 | 1085.68 | 0.97 | 6.10E-08 | 8.83E-08 | 1.13E-09 | 4.67E-09 | 1.39E-08 | 1.69E-05 | 3.25E-06 | 1.34E-06 | 7.72E-07 | 1.64E-06 | n.a. | n.a. | 9.83E-07 | 2.42E-06 | n.a. | n.a. | 3.36E-06 | 1.76E-06 | n.a. |
| 14 | 15/05/2012 | 1087.72 | 0.97 | 1.08E-07 | 6.55E-08 | 3.47E-09 | 1.59E-08 | 1.62E-08 | 2.59E-06 | 2.37E-06 | 1.12E-06 | 6.18E-07 | 2.39E-05 | n.a. | 2.10E-07 | 8.29E-07 | 1.50E-06 | 1.86E-07 | n.a. | 1.57E-06 | 5.81E-06 | 1.47E-07 |
| 15 | 19/05/2012 | 1051.59 | 1.00 | 2.33E-08 | 7.94E-08 | 7.97E-10 | 4.59E-09 | 1.49E-08 | 1.63E-05 | 1.64E-06 | 1.34E-06 | 6.32E-07 | 4.84E-06 | n.a. | n.a. | 9.97E-07 | 2.65E-06 | 2.21E-07 | n.a. | 3.28E-06 | 1.60E-06 | n.a. |
| 16 | 23/05/2012 | 997.82 | 1.05 | 3.69E-08 | 3.89E-08 | 5.83E-09 | 8.47E-09 | 1.75E-08 | 3.58E-06 | 1.56E-06 | 1.14E-06 | 6.74E-07 | 1.32E-06 | n.a. | 2.07E-07 | 8.23E-07 | 1.68E-06 | 1.89E-07 | n.a. | 1.54E-06 | 1.90E-06 | 1.45E-07 |
| 17 | 27/05/2012 | 1091.42 | 0.96 | 4.24E-08 | 9.03E-08 | 6.34E-10 | 1.08E-09 | 1.29E-08 | 4.07E-05 | 3.37E-06 | 3.07E-06 | 9.59E-07 | 4.81E-06 | n.a. | n.a. | 1.00E-06 | 5.00E-06 | 2.16E-07 | n.a. | 3.35E-06 | 2.42E-06 | n.a. |
| 18 | 31/05/2012 | 1040.93 | 1.01 | 1.09E-07 | 5.43E-08 | 3.66E-09 | 7.10E-09 | 1.57E-08 | 2.55E-06 | 1.82E-06 | 1.14E-06 | 5.70E-07 | 2.39E-05 | n.a. | 2.06E-07 | 8.18E-07 | 1.25E-06 | 1.86E-07 | n.a. | 1.50E-06 | 6.88E-06 | 1.48E-07 |
| 19 | 04/06/2012 | 1054.97 | 1.00 | 3.92E-08 | 2.14E-08 | 5.29E-10 | 2.26E-09 | 1.06E-08 | 1.66E-05 | 2.54E-06 | 1.28E-06 | 9.44E-07 | 4.77E-06 | n.a. | n.a. | 9.96E-07 | 2.43E-06 | 2.21E-07 | n.a. | 3.26E-06 | 1.60E-06 | 1.66E-06 |
| 20 | 08/06/2012 | 1092.18 | 0.96 | 3.57E-08 | 3.73E-08 | 3.38E-09 | 6.75E-09 | 1.07E-08 | 4.37E-06 | 1.86E-06 | 1.14E-06 | 5.89E-07 | 1.14E-06 | n.a. | 2.11E-07 | 8.37E-07 | 1.21E-05 | 1.86E-07 | n.a. | 2.17E-06 | 2.68E-06 | 1.51E-07 |
| 21 | 12/06/2012 | 998.35 | 1.05 | 3.66E-08 | 1.97E-08 | 3.85E-10 | 1.76E-09 | 1.21E-08 | 1.69E-05 | 1.98E-06 | 1.36E-06 | 7.37E-07 | 1.67E-06 | n.a. | n.a. | 9.98E-07 | 2.46E-06 | 2.20E-07 | n.a. | 3.31E-06 | 1.58E-06 | n.a. |
| 22 | 16/06/2012 | 1016.03 | 1.03 | 4.84E-08 | 3.67E-08 | 3.35E-09 | 6.72E-09 | 1.28E-08 | 2.82E-06 | 1.62E-06 | 1.12E-06 | 5.70E-07 | 1.23E-06 | n.a. | 2.09E-07 | 8.78E-07 | 1.60E-06 | 1.86E-07 | n.a. | 1.54E-06 | 2.33E-06 | 1.52E-07 |
| 23 | 20/06/2012 | 1013.04 | 1.04 | 3.02E-08 | 1.52E-08 | 4.55E-10 | 9.07E-10 | 1.06E-08 | 2.88E-05 | 1.98E-06 | 2.33E-06 | 1.10E-06 | 4.90E-06 | n.a. | n.a. | 9.85E-07 | 5.39E-06 | n.a. | n.a. | 3.32E-06 | 1.58E-06 | n.a. |
| 24 | 24/06/2012 | 1011.73 | 1.04 | 3.57E-08 | 3.61E-08 | 3.28E-09 | 6.52E-09 | 1.09E-08 | 2.49E-06 | n.a. | 1.17E-06 | 5.47E-07 | 1.02E-06 | n.a. | 2.05E-07 | 8.16E-07 | 1.20E-06 | 1.86E-07 | n.a. | n.a. | 5.13E-07 | n.a. |
| 25 | 28/06/2012 | 1026.34 | 1.02 | 2.40E-08 | 1.58E-08 | 4.84E-10 | 9.72E-10 | 1.55E-08 | 1.98E-05 | 2.68E-06 | 2.27E-06 | 1.34E-06 | 1.59E-06 | n.a. | n.a. | 1.00E-06 | 2.37E-06 | n.a. | n.a. | 3.33E-06 | 1.62E-06 | n.a. |
| 26 | 02/07/2012 | 1003.29 | 1.05 | 5.80E-08 | 3.88E-08 | 3.38E-09 | 6.53E-09 | 1.21E-08 | 2.71E-06 | 1.36E-06 | 1.11E-06 | 5.42E-07 | 1.23E-06 | n.a. | 2.11E-07 | 8.29E-07 | 1.22E-05 | n.a. | n.a. | 1.68E-06 | 1.48E-06 | 1.51E-07 |
| 27 | 06/07/2012 | 1037.11 | 1.01 | 2.07E-08 | 1.48E-08 | 3.41E-10 | 1.60E-09 | 1.08E-08 | 2.44E-05 | 2.75E-06 | 2.43E-06 | 1.72E-06 | n.a. | n.a. | n.a. | 1.00E-06 | 3.62E-06 | n.a. | n.a. | 3.37E-06 | 1.61E-06 | n.a. |
| 28 | 10/07/2012 | 1026.30 | 1.02 | 3.41E-08 | 3.74E-08 | 3.30E-09 | 7.45E-09 | 1.27E-08 | 4.23E-06 | 2.29E-06 | 1.16E-06 | 8.57E-07 | 1.25E-06 | n.a. | 2.05E-07 | 8.17E-07 | 1.21E-05 | n.a. | n.a. | 1.51E-06 | 8.60E-07 | 1.46E-07 |
| 29 | 14/07/2012 | 1029.54 | 1.02 | 2.66E-08 | 5.47E-08 | 4.49E-10 | 1.36E-09 | 1.26E-08 | 1.97E-05 | 2.22E-06 | 2.24E-06 | 1.34E-06 | 1.61E-06 | n.a. | n.a. | 1.01E-06 | 2.39E-06 | 2.19E-07 | n.a. | 3.35E-06 | 1.61E-06 | n.a. |
| 33 | 30/07/2012 | 1098.77 | 0.96 | 4.02E-08 | 3.65E-08 | 3.37E-09 | 6.54E-09 | 1.28E-08 | 3.24E-06 | 2.03E-06 | 1.12E-06 | 5.41E-07 | 1.22E-06 | n.a. | 2.06E-07 | 8.17E-07 | 1.31E-05 | 1.86E-07 | n.a. | 1.54E-06 | 1.02E-06 | 1.47E-07 |
| 34 | 03/08/2012 | 1075.06 | 0.98 | 2.02E-08 | n.a. | 4.25E-10 | 6.87E-10 | 9.47E-09 | 1.96E-05 | 2.41E-06 | 2.23E-06 | 1.27E-06 | 1.62E-06 | n.a. | n.a. | 1.02E-06 | 2.66E-06 | n.a. | n.a. | 3.37E-06 | 1.65E-06 | n.a. |
| 35 | 07/08/2012 | 1074.20 | 0.98 | 7.08E-08 | 3.74E-08 | 3.32E-09 | 6.57E-09 | 1.24E-08 | 2.45E-06 | 2.40E-06 | 1.13E-06 | 5.62E-07 | 1.25E-06 | n.a. | 2.05E-07 | 8.18E-07 | 1.30E-05 | 1.86E-07 | n.a. | 1.49E-06 | 7.87E-07 | 1.45E-07 |
| 36 | 11/08/2012 | 919.08 | 1.14 | 2.88E-08 | 1.47E-08 | 5.37E-10 | 1.20E-09 | 1.28E-08 | 2.09E-05 | 2.48E-06 | 2.28E-06 | 1.39E-06 | 1.61E-06 | n.a. | 1.42E-06 | 9.91E-07 | 2.38E-06 | n.a. | n.a. | 3.24E-06 | 1.63E-06 | n.a. |
| 37 | 15/08/2012 | 1088.64 | 0.96 | 3.30E-08 | 3.63E-08 | 3.33E-09 | 6.53E-09 | 1.11E-08 | 2.91E-06 | 2.40E-06 | 1.15E-06 | 5.63E-07 | 1.23E-06 | n.a. | 2.08E-07 | 8.17E-07 | 1.56E-06 | 1.87E-07 | n.a. | 1.49E-06 | 6.93E-07 | 1.45E-07 |

| Sample | Date | Volume (m^3^) | f ^a^ | Al | Fe | Cu | Mn | Zn | Na | K | Mg | Ca | NH_4_^+^ | Acetate | Formate | Cl^-^ | NO_2_^-^ | NO_3_^-^ | SO_4_^2-^ | Malonate | Oxalate | PO_4_^3-^ |
| --- | --- | --- | --- | --- | --- | --- | --- | --- | --- | --- | --- | --- | --- | --- | --- | --- | --- | --- | --- | --- | --- | --- |
| 38 | 19/08/2012 | 1057.32 | 0.99 | 3.03E-08 | 1.61E-08 | 4.34E-10 | 1.84E-09 | 1.26E-08 | 2.00E-05 | 2.43E-06 | 2.24E-06 | 1.63E-06 | n.a. | n.a. | n.a. | 1.02E-06 | 3.91E-06 | 2.22E-07 | n.a. | 3.37E-06 | 1.63E-06 | n.a. |
| 39 | 23/08/2012 | 1074.06 | 0.98 | 3.38E-08 | 3.63E-08 | 3.33E-09 | 6.67E-09 | 1.18E-08 | 3.91E-06 | 1.35E-06 | 1.15E-06 | 6.16E-07 | 1.23E-06 | n.a. | 2.05E-07 | 8.17E-07 | 1.29E-05 | 1.86E-07 | n.a. | 1.49E-06 | 8.23E-07 | n.a. |
| 40 | 27/08/2012 | 1073.32 | 0.98 | 8.75E-08 | 1.94E-08 | 3.80E-09 | 2.17E-09 | 1.17E-08 | 1.94E-05 | 2.15E-06 | 2.32E-06 | 1.40E-06 | 4.88E-06 | n.a. | 1.34E-06 | 1.04E-06 | 2.93E-06 | 2.19E-07 | n.a. | 4.51E-06 | 1.60E-06 | n.a. |
| 41 | 31/08/2012 | 1082.66 | 0.97 | 3.36E-08 | 3.60E-08 | 4.32E-09 | 6.66E-09 | 9.46E-09 | 4.06E-06 | 2.00E-06 | 1.20E-06 | 5.81E-07 | 1.26E-06 | n.a. | 2.05E-07 | 8.17E-07 | 1.54E-05 | 1.86E-07 | n.a. | 1.52E-06 | 1.44E-06 | 1.45E-07 |
| 42 | 04/09/2012 | 1062.70 | 0.99 | 1.91E-08 | 1.48E-08 | 3.75E-10 | 9.46E-10 | 1.20E-08 | 2.10E-05 | 3.14E-06 | 2.76E-06 | 1.76E-06 | n.a. | n.a. | n.a. | 1.01E-06 | 4.76E-06 | n.a. | n.a. | 3.34E-06 | 1.62E-06 | n.a. |

^a^ multiplicative correction factor used to correct the concentrations in order to be all referred to the average volume of sampled air: $f_{i}=\bar{V}/{V_{i}}$, $V_{i}$ = volume of air collected, $\bar{V}$ = average volume of air collected. Not available data are indicated as “n.a.”.

Table S7. Limits of detection (LOD) of the techniques used for the analysis of the components of the soluble fraction of the Arctic PM_10_.

| **Component** | **Technique** | **LOD (mol L^-1^)** |  | **Component** | **Technique** | **LOD (mol L^-1^)** |
| --- | --- | --- | --- | --- | --- | --- |
| Al | SF-ICP-MS | 1.15×10^-10^ |  | Acetate | Anionic IC | 2.08×10^-7^ |
| Fe | SF-ICP-MS | 1.43×10^-10^ |  | Formate | Anionic IC | 6.13×10^-8^ |
| Cu | SF-ICP-MS | 8.42×10^-11^ |  | Cl^-^ | Anionic IC | 2.31×10^-6^ |
| Mn | SF-ICP-MS | 5.66×10^-11^ |  | NO_2_^-^ | Anionic IC | 1.82×10^-7^ |
| Zn | SF-ICP-MS | 1.66×10^-10^ |  | NO_3_^-^ | Anionic IC | 1.44×10^-6^ |
| Na | ICP-OES | 7.75×10^-7^ |  | SO_4_^2-^ | Anionic IC | 3.92×10^-7^ |
| K | ICP-OES | 4.73×10^-7^ |  | Malonate | Anionic IC | 1.47×10^-7^ |
| Mg | ICP-OES | 3.39×10^-8^ |  | Oxalate | Anionic IC | 1.47×10^-7^ |
| Ca | ICP-OES | 8.69×10^-8^ |  | PO_4_^3-^ | Anionic IC | 5.57×10^-6^ |
| NH_4_^+^ | Cationic IC | 7.61×10^-7^ |  |  |  |  |

Table S8. Errors of certified reference material (CRM) analysis for some components.

| **Component** | **CRM** | **Error (%)** |
| --- | --- | --- |
| Na | ERM-CZ110 | 7.83 |
| K | ERM-CZ110 | 3.88 |
| Mg | ERM-CZ110 | 3.29 |
| Ca | ERM-CZ110 | -2.80 |
| NH_4_^+^ | TraceCERT Multi Cation Standard* | 1.6 |
| Cl^-^ | TraceCERT Multi anion* | 0.17 |
| NO_3_^-^ | TraceCERT Multi anion* | -1.15 |
| SO_4_^2-^ | TraceCERT Multi anion* | 1.68 |
| PO_4_^3-^ | TraceCERT Multi anion* | -26.99 |

* Only instrumental analysis

Table S9. Concentration (mol L^-1^) of the components reported in this work and found in literature regard precipitations collects in various part of the Arctic.

| Reference | This work | [13] | [14] | [15] | | [16] | [17] | [18] | [19] | [20] |
| --- | --- | --- | --- | --- | --- | --- | --- | --- | --- | --- |
| Type of sample | Solubilized PM | snowfall | Fresh snow | rain | snowfall | Fresh snow | rain | Rain and snowfall | rain | rain |
| Al | 5.2×10^-7^ | 6.3×10^-7^ | 2.7×10^-7^ |  |  |  |  |  |  |  |
| Fe | 2.2×10^-7^ |  | 1.9×10^-7^ |  |  |  |  | 1.4×10^-6^ |  |  |
| Cu | 9.3×10^-9^ | 7.7×10^-8^ | 7.9×10^-10^ |  |  |  |  | 8.5×10^-8^ |  |  |
| Mn | 3.7×10^-8^ | 5.3×10-8 | 1.2×10^-8^ |  |  |  |  | 1.5×10^-7^ |  |  |
| Zn | 1.0×10^-7^ | 1.2×10-7 |  |  |  |  |  | 6.4×10^-7^ |  |  |
| Na | 2.3×10^-4^ | 1.6×10-7 | 4.8×10^-6^ | 8.9×10^-5^ | 3.0×10^-4^ | 6.7×10^-5^ |  | 1.8×10^-4^ | 8.3×10^-5^ | 4.6×10^-6^ |
| K | 6.1×10^-6^ | 1.6×10-7 | 3.9×10^-7^ | 1.0×10^-7^ | 1.7×10^-5^ | 2.3×10^-6^ |  | 1.6×10^-5^ | 2.9×10^-5^ | 1.6×10^-6^ |
| Mg | 3.0×10^-5^ | 8.0×10-8 | 1.8×10^-6^ | 2.5×10^-5^ | 7.9×10^-5^ | 9.2×10^-6^ | 2.8×10^-4^ | 4.3×10^-5^ | 9.9×10^-5^ | 1.8×10^-6^ |
| Ca | 1.3×10^-5^ | 2.4×10-8 | 4.8×10^-6^ | 7.8×10^-6^ | 1.4×10^-5^ | 4.1×10^-6^ | 6.8×10^-5^ | 3.2×10^-5^ | 5.9×10^-5^ | 4.2×10^-6^ |
| NH_4_^+^ | 3.3×10^-5^ |  | 6.9×10^-7^ |  |  |  | 3.1×10^-5^ | 1.0×10^-5^ | 4.2×10^-5^ | 5.1×10^-6^ |
| Ac | 5.6×10^-7^ |  | 3.4×10^-7^ |  |  | 2.5×10^-6^ |  |  |  | 7.0×10^-6^ |
| For | 1.1×10^-5^ |  | 2.4×10^-7^ |  |  | 1.2×10^-6^ |  |  |  | 7.9×10^-6^ |
| Cl^-^ | 1.8×10^-4^ |  | 7.0×10^-6^ | 4.6×10^-5^ | 6.0×10^-5^ | 1.7×10^-4^ | 1.4×10^-3^ | 3.0×10^-4^ | 9.1×10^-5^ | 9.4×10^-6^ |
| NO_3_^-^ | 3.0×10^-5^ |  | 2.5×10^-6^ | 1.9×10^-6^ | 7.1×10^-7^ | 5.2×10^-6^ | 1.7×10^-5^ | 6.2×10^-6^ | 1.2×10^-5^ | 3.7×10^-6^ |
| SO_4_^2-^ | 7.0×10^-5^ |  | 3.1×10^-6^ | 8.2×10^-6^ | 3.3×10^-6^ | 6.6×10^-6^ | 1.9×10^-4^ | 9.8×10^-6^ | 1.6×10^-4^ | 8.6×10^-6^ |
| Mal | 6.9×10^-7^ |  |  |  |  |  |  |  |  |  |
| Ox | 2.7×10^-6^ |  |  |  |  | 4.5×10^-8^ |  |  |  | 1.4×10^-6^ |
| PO_4_^3-^ | 1.6×10^-5^ |  |  |  |  |  |  |  |  | 5.0×10^-6^ |
| pH | 6.4 |  |  | 5.5 | 5.7 |  | 4.7 | 5.7 | 6.1 | 4.7 |

Table S10. Pearson’s correlation coefficients. The signature coefficients at a significance level of 0.05 are shown in italic.

| Component | Al | Fe | Cu | Mn | Zn | Na | K | Mg | Ca | NH_4_^+^ | Acetate | Formate | Cl^-^ | NO_2_^-^ | NO_3_^-^ | SO_4_^2-^ | Malonate | Oxalate | PO_4_^3-^ |
| --- | --- | --- | --- | --- | --- | --- | --- | --- | --- | --- | --- | --- | --- | --- | --- | --- | --- | --- | --- |
| Al | 1.00 |  |  |  |  |  |  |  |  |  |  |  |  |  |  |  |  |  |  |
| Fe | *0.95* | 1.00 |  |  |  |  |  |  |  |  |  |  |  |  |  |  |  |  |  |
| Cu | *0.73* | 0.65 | 1.00 |  |  |  |  |  |  |  |  |  |  |  |  |  |  |  |  |
| Mn | *0.82* | 0.77 | 0.57 | 1.00 |  |  |  |  |  |  |  |  |  |  |  |  |  |  |  |
| Zn | *0.71* | 0.69 | 0.52 | 0.68 | 1.00 |  |  |  |  |  |  |  |  |  |  |  |  |  |  |
| Na | -0.11 | -0.09 | 0.09 | -0.09 | -0.11 | 1.00 |  |  |  |  |  |  |  |  |  |  |  |  |  |
| K | 0.10 | 0.09 | 0.30 | 0.10 | 0.01 | *0.93* | 1.00 |  |  |  |  |  |  |  |  |  |  |  |  |
| Mg | -0.08 | -0.05 | 0.14 | -0.04 | -0.10 | 0.99 | *0.96* | 1.00 |  |  |  |  |  |  |  |  |  |  |  |
| Ca | 0.34 | 0.33 | 0.35 | *0.59* | 0.27 | *0.64* | *0.72* | *0.70* | 1.00 |  |  |  |  |  |  |  |  |  |  |
| NH_4_^+^ | *0.91* | *0.86* | *0.71* | *0.76* | *0.76* | -0.11 | 0.09 | -0.07 | 0.31 | 1.00 |  |  |  |  |  |  |  |  |  |
| Acetate | 0.15 | 0.03 | 0.19 | -0.01 | 0.04 | 0.09 | 0.23 | 0.14 | 0.01 | 0.30 | 1.00 |  |  |  |  |  |  |  |  |
| Formate | 0.18 | 0.06 | 0.21 | 0.02 | 0.08 | 0.11 | 0.24 | 0.16 | 0.04 | 0.33 | *0.99* | 1.00 |  |  |  |  |  |  |  |
| Cl^-^ | *-0.42* | *-0.40* | -0.18 | -0.32 | *-0.38* | *0.83* | *0.74* | *0.81* | *0.43* | -0.42 | -0.05 | -0.06 | 1.00 |  |  |  |  |  |  |
| NO_2_^-^ | 0.08 | 0.09 | 0.19 | 0.14 | 0.14 | 0.30 | 0.36 | 0.35 | *0.39* | 0.03 | -0.08 | -0.10 | 0.04 | 1.00 |  |  |  |  |  |
| NO_3_^-^ | 0.16 | 0.03 | 0.21 | -0.01 | 0.04 | 0.11 | 0.25 | 0.16 | 0.03 | 0.30 | *0.99* | *0.97* | -0.05 | -0.04 | 1.00 |  |  |  |  |
| SO_4_^2-^ | *0.67* | *0.71* | *0.66* | *0.49* | *0.50* | *0.43* | *0.57* | *0.49* | *0.55* | *0.66* | 0.31 | 0.34 | -0.06 | *0.45* | 0.31 | 1.00 |  |  |  |
| Malonate | 0.28 | 0.15 | 0.25 | 0.09 | 0.15 | 0.16 | 0.23 | 0.18 | 0.07 | *0.40* | *0.91* | *0.93* | -0.10 | -0.19 | *0.90* | *0.39* | 1.00 |  |  |
| Oxalate | *0.37* | 0.22 | 0.34 | 0.28 | 0.29 | 0.23 | 0.29 | 0.25 | 0.29 | *0.48* | *0.68* | *0.70* | -0.12 | 0.03 | *0.71* | *0.45* | *0.84* | 1.00 |  |
| PO_4_^3-^ | 0.14 | 0.01 | 0.19 | -0.03 | 0.02 | 0.08 | 0.23 | 0.13 | 0.00 | 0.29 | *1.00* | *0.98* | -0.05 | -0.05 | *0.99* | 0.29 | *0.89* | *0.66* | 1.00 |


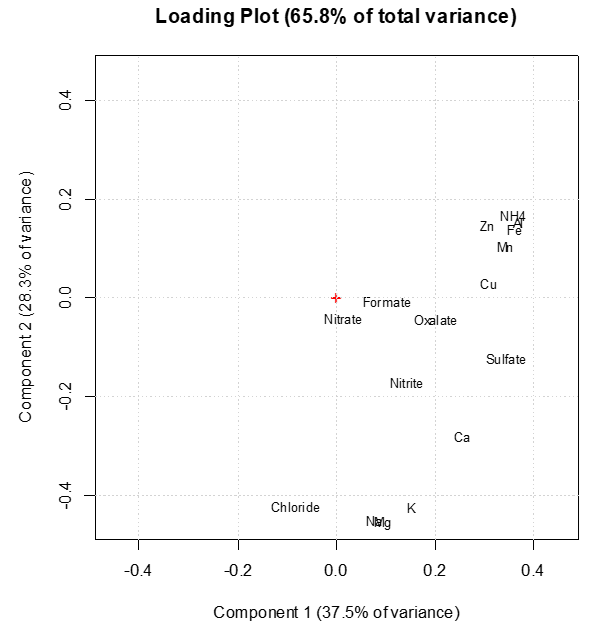


**Fig S1** Loading plot for the first two principal components


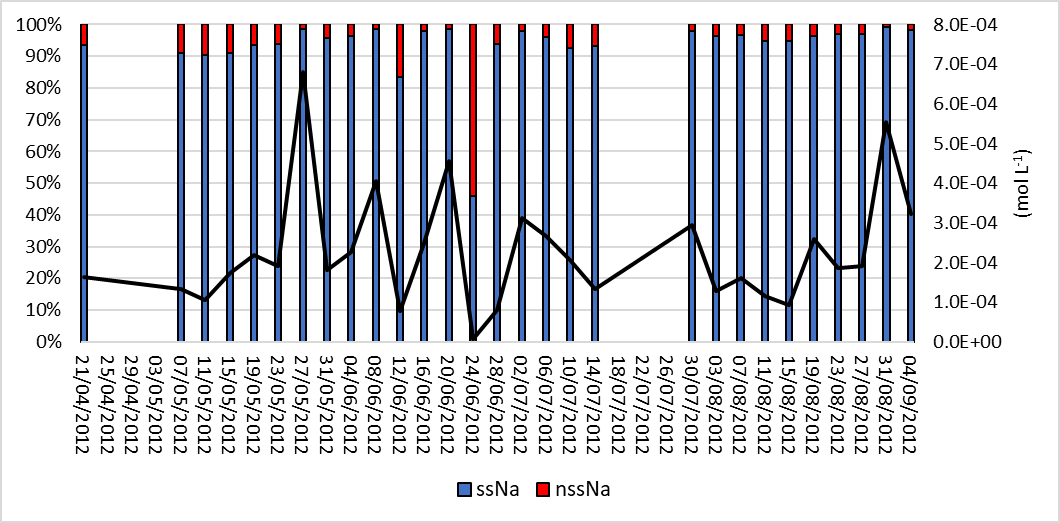


(a)


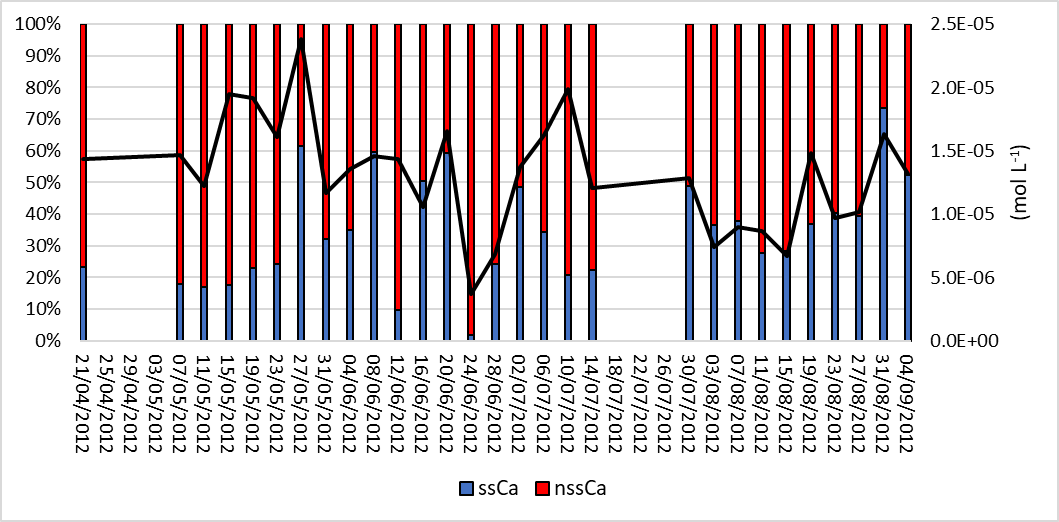


(b)


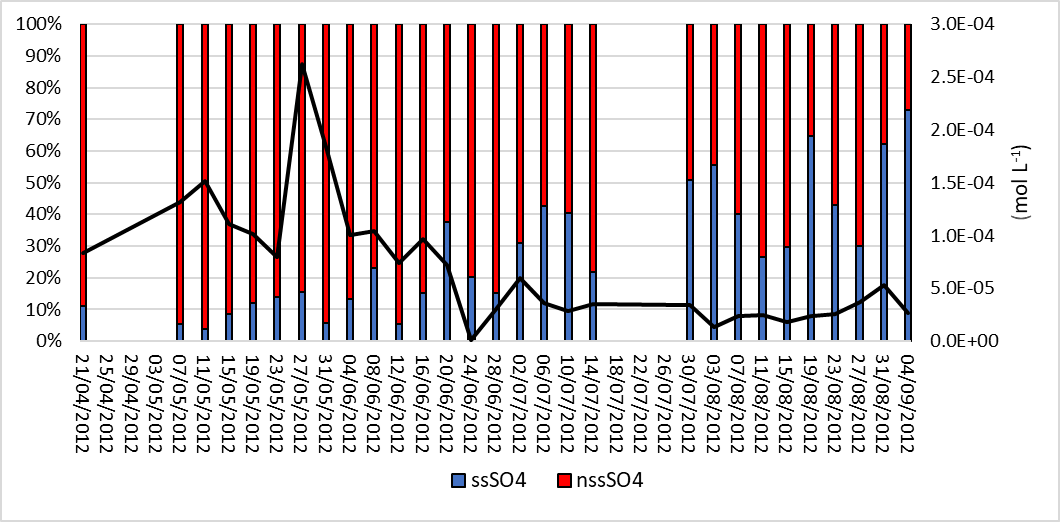


(c)

**Fig S2** Temporal trends of (a) Na, (b) Ca, and (c) SO_4_^2-^ concentrations (black lines) and their percentage distribution between the sea salt (blue) and non-sea salt (red) contribution


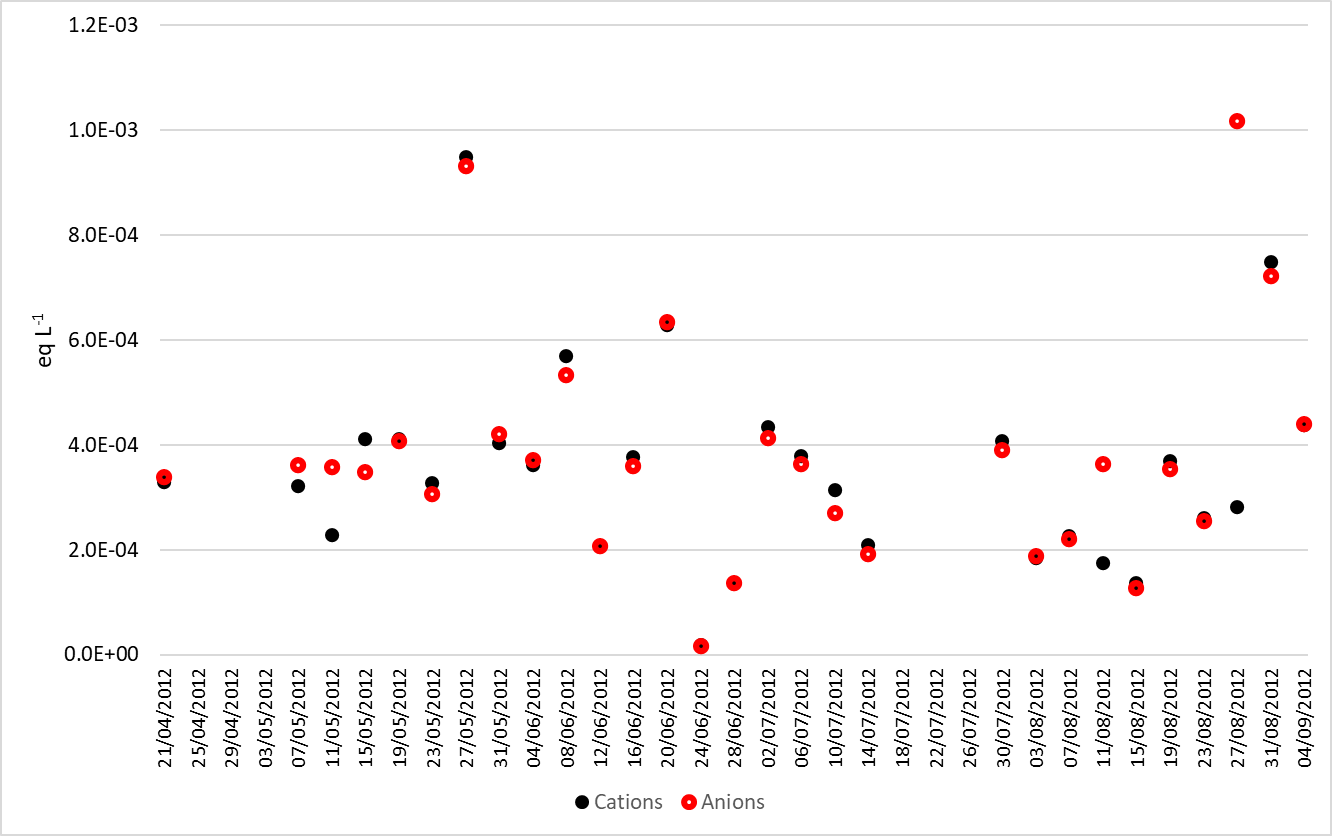


**Fig S3** Cation (black) and anion (red) concentration (as eq L^-1^) for the solutions obtained by the extraction of the soluble fraction of PM_10_ samples collected at Ny-Ålesund during the spring-summer campaign of 2012


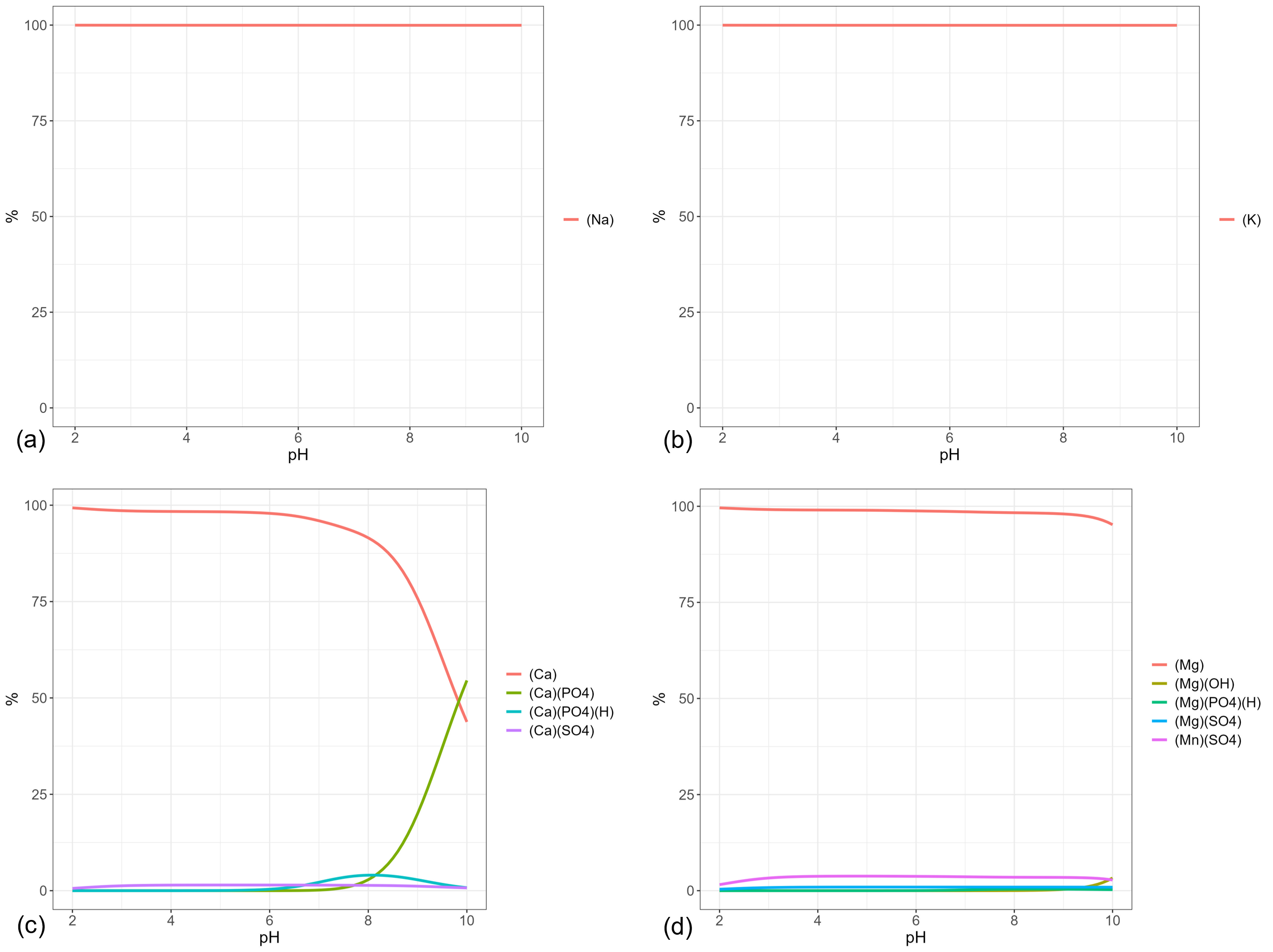


**Fig S4** Mean species distribution diagrams of (a) Na, (b) K, (c) Ca, and (d) Mg as a function of pH for the soluble fraction of 29 Arctic PM_10_ samples collected in 2012


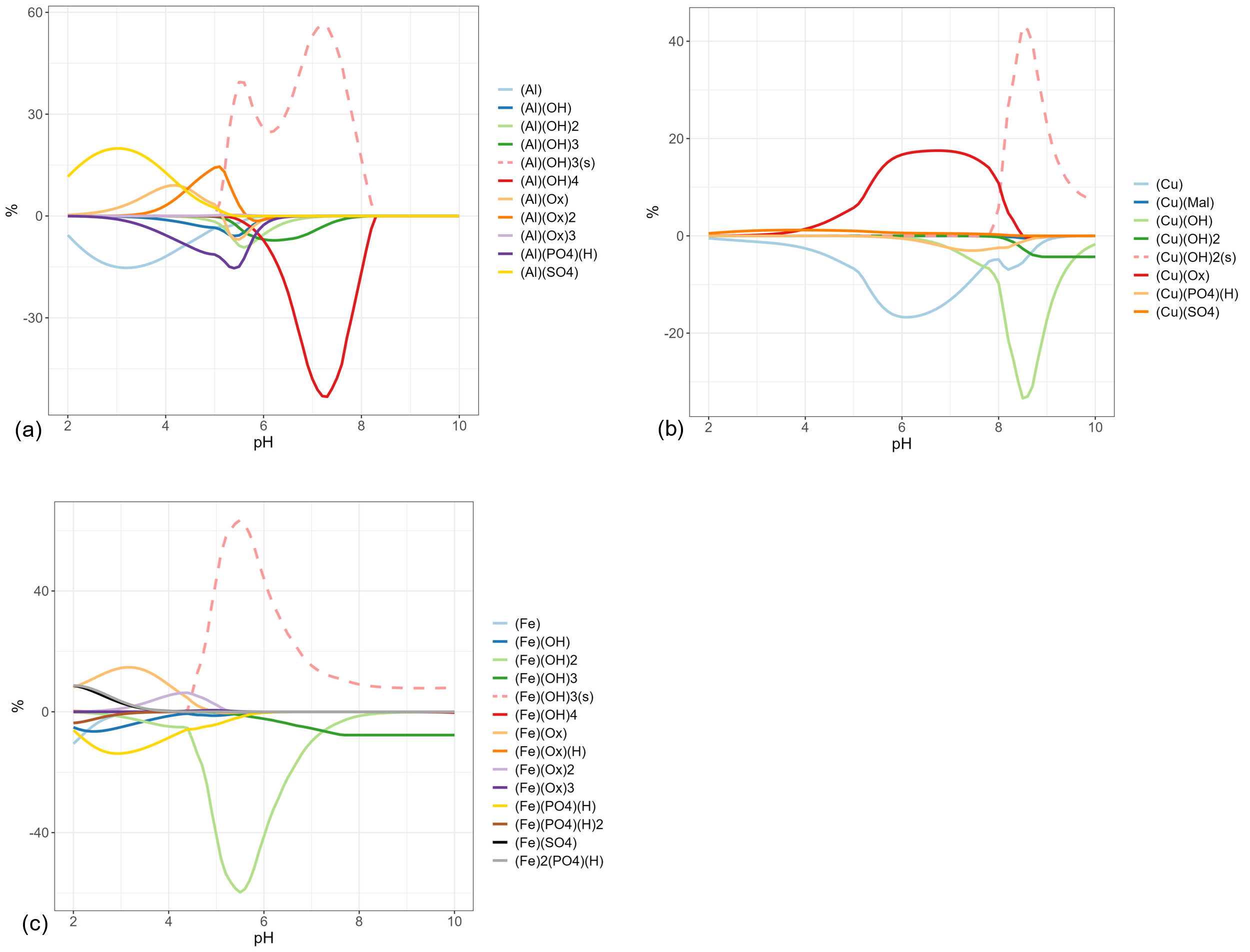


**Fig S5** Differences between the mean spring and summer speciation diagrams for (a) Al, (b) Cu, and (d) Fe occurring in the soluble fraction of the Arctic PM10 samples collected in 2012


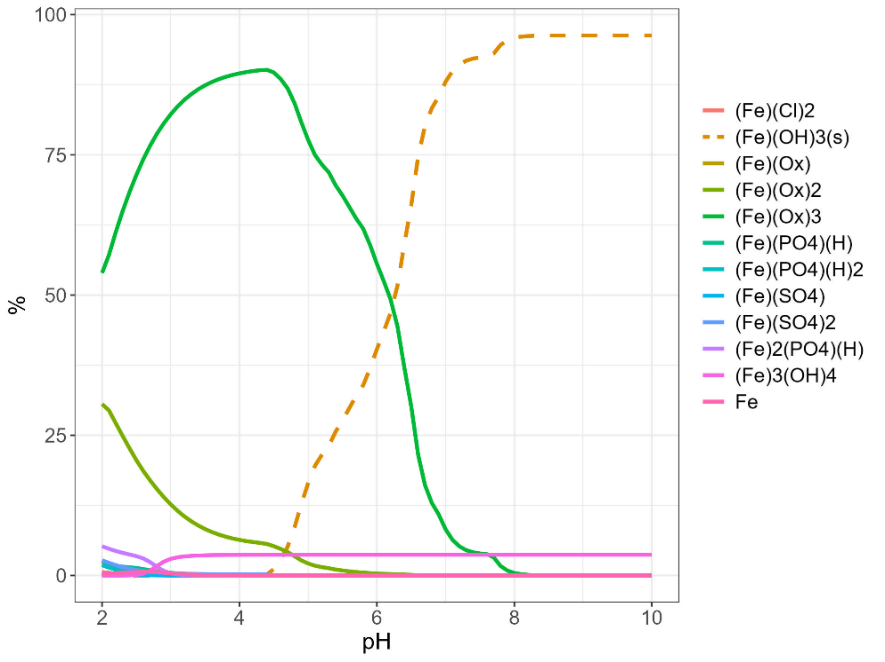


**Fig S6** Mean species distribution diagrams of Fe occurring in the soluble fraction of Arctic PM_10_ collected in 2012 as a function of pH, for the component concentration increased by a factor of 10000


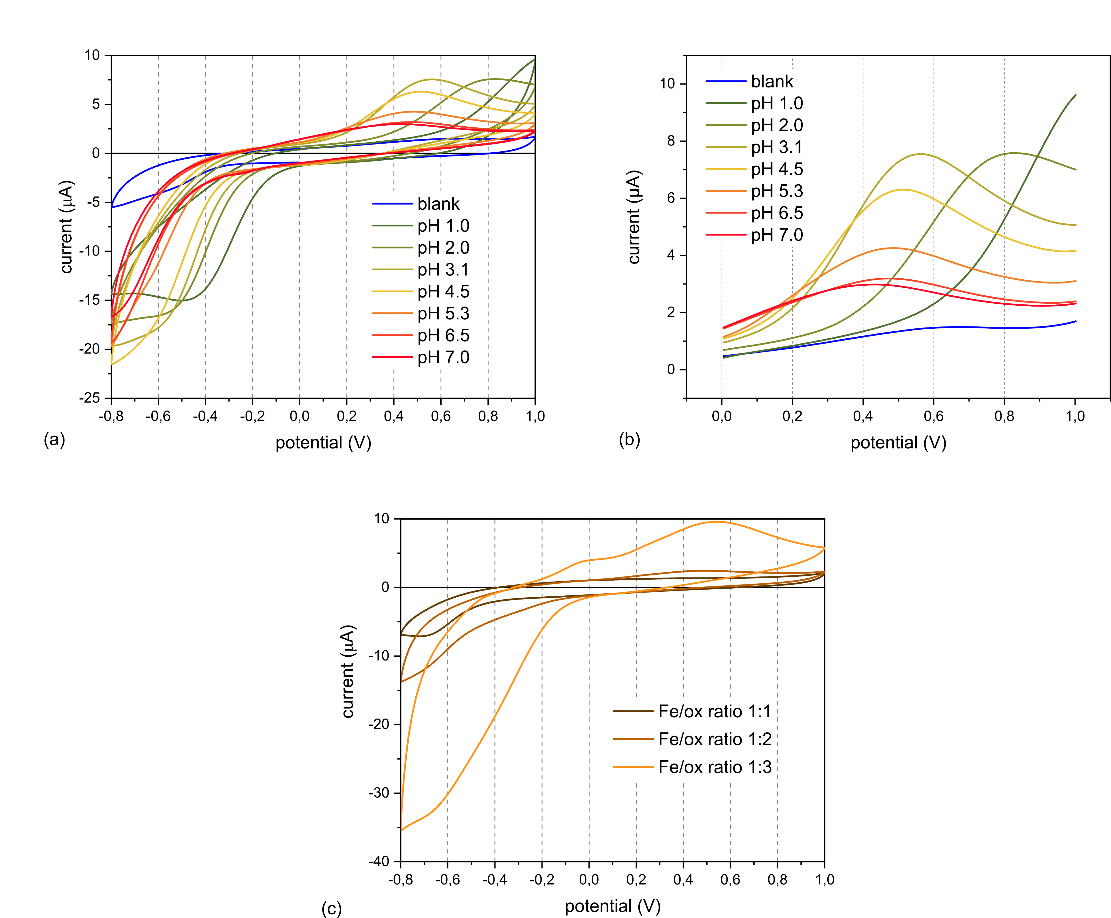


**Fig S7** Cyclic voltammetry scans of Fe^3+^-Ox system. WE: GCE; CE: Pt wire; RE: Ag/AgCl. Medium in NaCl 1.0 mol L^-1^ (a) C_Fe_^3+^ = 0.002 mol L^-1^; C_ox_ = 0.02 mol L^-1^ varing pH of solution; (b) same scans of (a) but focusing only on the oxidation peaks at positive potentials; (c) pH = 5.5; C_Fe_^3+^ = 0.002 mol L^-1^ varing the concentration of oxalate.


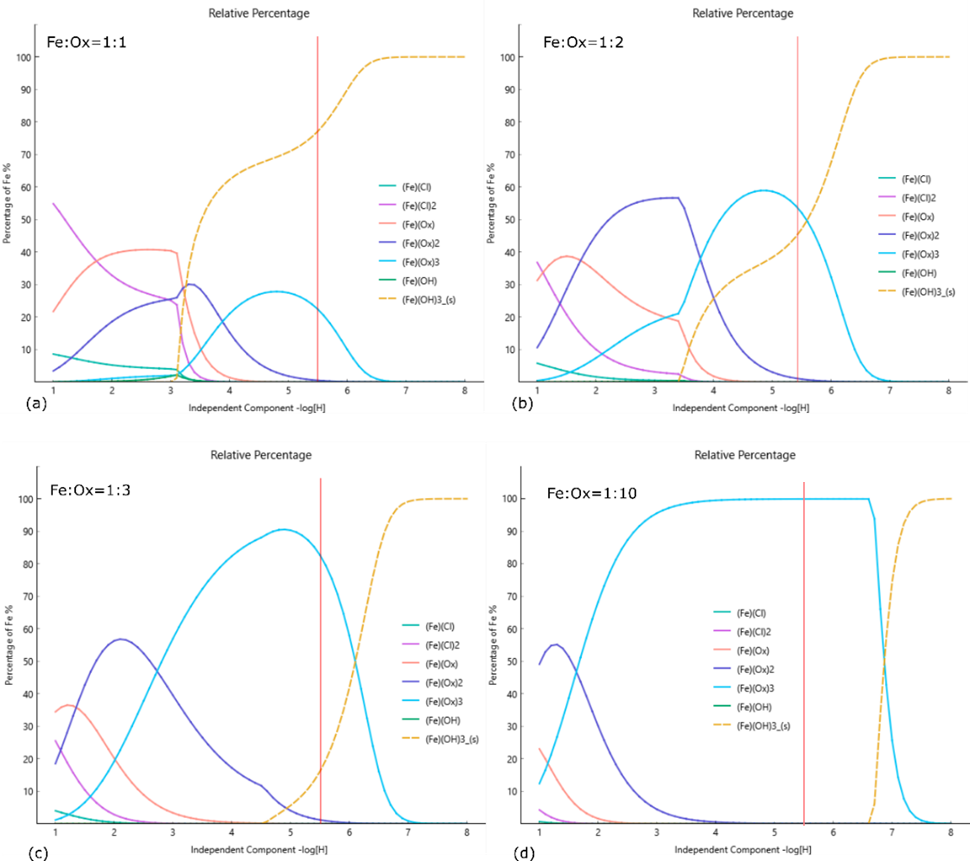


**Fig S8** Species distribution diagram for the system Fe-Ox at high concentration of components (C_NaCl_ = 1 mol L^-1^, C_Fe_^3+^ = 0.002 mol L^-1^, C_NO3_^2-^ = 0.006 mol L^-1^) varying the concentration of oxalate (as potassium oxalate): (a) C_Ox_ = 0.002 mol L^-1^; (b) C_Ox_ = 0.004 mol L^-1^; c) C_Ox_ = 0.006 mol L^-1^; (d) C_Ox_ = 0.02 mol L^-1^. The red line marks pH 5.5

References

1. Casale A, Daniele PG, De Robertis A, Sammartano S. Ionic strength dependence of formation constants. Part XI. An analysis of literature data on carboxylate ligand complexes. Ann Chim. 1988;78:249–260

2. Goldberg RN, Kishore N, Lennen RM. Thermodynamic Quantities for the Ionization Reactions of Buffers. J Phys Chem Ref Data. 2002;31:231. https://doi.org/10.1063/1.1416902

3. Daniele PG, De Robertis A, De Stefano C, Gianguzza A, Sammartano S. Salt effects on the protonation of ortho-phosphate between 10 and 50°C in aqueous solution. A complex formation model. J Solution Chem. 1991;20:495–515. https://doi.org/10.1007/BF00650805

4. Daniele PG, De Robertis A, De Stefano C, Sammartano S, Rigano C. On the possibility of determining the thermodynamic parameters for the formation of weak complexes using a simple model for the dependence on ionic strength of activity coefficients: Na. J Chem Soc Dalt Trans. 1985;2353–2361

5. Accademic Software. SC-Database. The IUPAC stability constant database

6. De Robertis A, Rigano C, Sammartano S, Zerbinati O. Ion association of Cl- with Na+, K+, Mg2+ and Ca2+ in aqueous solution at 10 ≤ T ≤ 45 ° C and 0 ≤ I ≤ 1 mol l-1. A literature data analysis. Thermochim Acta. 1987;115:241–248. https://doi.org/10.1016/0040-6031(87)88370-3

7. Millero FJ, Yao W, Aicher J. The speciation of Fe(II) and Fe(III) in natural waters. Mar Chem. 1995;50:21–39. https://doi.org/10.1016/0304-4203(95)00024-L

8. Chughtai A, Marshall R, Nancollas GH. Complexes in calcium phosphate solutions. J Phys Chem. 1968;72:208–211. https://doi.org/10.1021/j100847a039

9. Smith RM, Martell AE. Critical Stability Constants. Plenum Press, New York

10. Izatt BRM, Eatough D, Christensen JJ, Bartholomew CH. Inorg. Phys. Theor. 47. 1967;45–47

11. De Robertis A, Di Giacomo P, Foti C. Ion-selective electrode measurements for the determination of formation constants of alkali and alkaline earth metals with low-molecular-weight ligands. Anal Chim Acta. 1995;300:45–51. https://doi.org/10.1016/0003-2670(94)00421-H

12. Paris R, Desboeufs K V. Effect of atmospheric organic complexation on iron-bearing dust solubility. Atmos Chem Phys. 2013;13:4895–4905. https://doi.org/10.5194/acp-13-4895-2013

13. Lee D-I, Kikuchi K, Taniguchi T. Chemical Compositions of Aerosol Particles and Snowfalls at Alta, Northern Norway. Environ Sci Hokkaido Univ. 1989;12:169–178

14. MacDonald KM, Sharma S, Toom D, Chivulescu A, Hanna S, Bertram AK, Platt A, Elsasser M, Huang L, Tarasick D, Chellman N, McConnell JR, Bozem H, Kunkel D, Duan Lei Y, Evans GJ, Abbatt JPD. Observations of atmospheric chemical deposition to high Arctic snow. Atmos Chem Phys. 2017;17:5775–5788. https://doi.org/10.5194/acp-17-5775-2017

15. Hou S, Qin D. Chemical characteristics of summer precipitation at Point Barrow, Arctic Alaska. Chinese J Polar Sci. 1997;8:1–7

16. Hegg DA, Warren SG, Grenfell TC, Doherty SJ, Larson T V., Clarke AD. Source attribution of black carbon in arctic snow. Environ Sci Technol. 2009;43:4016–4021. https://doi.org/10.1021/es803623f

17. Lide D. Aqueous Solubility of Inorganic Compounds at Various Temperatures. CRC Handb Chem Phys. 2005;1–6

18. Svistov PF, Pershina NA, Pavlova MT, Polishchuk AI, Semenets ES. Chemical composition of Russian Arctic precipitation in 2007-2015. Russ Meteorol Hydrol. 2017;42:314–318. https://doi.org/10.3103/S1068373917050065

19. Svistov PF, Talash AS, Semenets ES. Air Pollution and Self-Purification by Precipitation in the Russian Arctic. Russ J Gen Chem. 2017;87:3173–3182. https://doi.org/10.1134/S1070363217130114

20. Talbot RW, Vijgen AS, Harriss RC. Soluble species in the Arctic summer troposphere: acidic gases, aerosols, and precipitation. J Geophys Res. 1992; 97. https://doi.org/10.1029/91jd00118
